# Three hospitalized non-critical COVID-19 subphenotypes and change in intubation or death over time: A latent class analysis with external and longitudinal validation

**DOI:** 10.1371/journal.pone.0316434

**Published:** 2025-03-19

**Authors:** William S. Stringer, Amy S. Labar, Joshua D. Geleris, Evan V. Sholle, David A. Berlin, Claire M. McGroder, Matthew J. Cummings, Max R. O’Donnell, Haoyang Yi, Xuehan Yang, Ying Wei, Edward J. Schenck, Matthew R. Baldwin

**Affiliations:** 1 Division of Pulmonary, Allergy, and Critical Care, Columbia University Vagelos College of Physicians and Surgeons, New York, New York, United States of America; 2 Department of Medicine, University of Pittsburgh Medical Center, Pittsburgh, Pennsylvania, United States of America; 3 Division of Pulmonary and Critical Care Medicine, Weill Cornell University School of Medicine, New York, New York, United States of America; 4 Department of Biostatistics, Columbia Mailman School of Public Health, New York, New York, United States of America; Stellenbosch University, SOUTH AFRICA

## Abstract

**Background:**

There are two subphenotypes of COVID-19 acute respiratory distress syndrome with differential responses to corticosteroids, but whether similar subphenotypes of hospitalized non-critical COVID-19 patients exist remains unknown.

**Objective:**

To identify and validate non-critical COVID-19 subphenotypes at hospital admission that may elucidate pathobiology and facilitate heterogeneity-of-treatment effect analyses of clinical trials with non-critical COVID-19 patients.

**Methods:**

We conducted a multi-center retrospective cohort study of adults hospitalized with COVID-19 who were not intubated or did not die within 24 hours of admission. We derived and externally and longitudinally validated subphenotypes during the wild-type and delta severe-acute-respiratory-syndrome-coronavirus-2 (SARS-CoV2) waves via latent class analysis using clinical and laboratory data at hospital admission. We trained XGBoost machine learning models to predict subphenotype.

**Results:**

We analyzed data for 4,827 hospitalized non-critical COVID-19 patients: 2,077 wild-type wave Columbia University Medical Center (CUMC) and affiliate hospital derivation cohort patients; 1,214 wild-type wave Cornell Medical Center and affiliate hospital external validation cohort patients; and 1,536 delta wave CUMC and affiliate hospital longitudinal validation cohort patients. A three-class latent class model best fit each cohort identifying subphenotypes that were low-inflammatory, intermediate-inflammatory, and high-inflammatory with fibrinolysis, with increasing 90-day risk of intubation or death across subphenotypes in the wild-type wave. However, in the delta wave, the intermediate-inflammatory subphenotype had the lowest 90-day risk of intubation or death. XGBoost model area under the receiver-operating-curve was 0.96 in the testing dataset, and biomarkers of inflammation and cardiorenal dysfunction were the strongest predictors of subphenotype.

**Conclusion:**

We identified three hospitalized non-critical COVID-19 subphenotypes that persisted through the wild-type and delta SARS-CoV2 waves. The intermediate-inflammatory subphenotype had the greatest relative improvement in intubation and survival over time with the standardized use of corticosteroids and other interventions. Our machine learning model can facilitate heterogeneity-of-treatment effect analyses of clinical trials of adults hospitalized with non-critical COVID-19.

## Introduction

Identification of hypo- and hyper-inflammatory subphenotypes in non-coronavirus disease-19 (non-COVID-19) and COVID-19 acute respiratory distress syndrome (ARDS) has facilitated post-hoc heterogeneity-of-treatment effect analyses that have revealed differential responses to positive end expiratory pressure (PEEP) [1], intravenous fluids [2], simvastatin [3], and possibly, corticosteroids [4]. These studies have influenced how we consider treatment of ARDS patients at the bedside and predictive and prognostic enrichment of future ARDS trials [5]. However, subphenotyping after the onset of critical illness has limited value in identifying subgroups of non-critical acute lung injury (ALI) patients who are most likely to benefit from therapeutics aimed at inhibiting progression of disease.

Several trials, including the Accelerating COVID-19 Therapeutic Interventions and Vaccines (ACTIV)-1 and 3a trials [6,7], the Anti-Coronavirus Therapies (ACT) COVID-19 trials [8], and Randomised Evaluation of COVID-19 Therapy (RECOVERY) trials [9], as well as other trials, enrolled adults with non-critical COVID-19 and found no benefit from most of the drugs tested to prevent progression of COVID-19. Prior subphenotyping studies of patients hospitalized with non-critical COVID-19 have not led to post-hoc heterogeneity-of-treatment effect analyses of these trials for several reasons. Subphenotyping study patients are not always representative of trial patients, additionally including those discharged from the emergency department (ED) or who were critically-ill at admission [10–12]. Exogenous variables, such as socio-demographics and symptoms, that do not biologically define subphenotypes were sometimes used as class defining variables [10,13]. Subphenotypes were derived in the wild-type severe acute respiratory syndrome coronavirus-2 (SARS-CoV2) wave and never longitudinally validated in the delta SARS-CoV2 wave when many trial patients were enrolled [10–14]. Lastly, none of the prior subphenotyping studies have a usable subphenotype prediction model.

To facilitate post-hoc heterogeneity-of-treatment effect analyses of trials with hospitalized non-critical COVID-19 patients, we selected patients meeting these trials’ general eligibility criteria of adults who were not critically ill within 24 hours after ED presentation. We selected variables at hospital admission that were used in prior studies to identify non-COVID-19 ARDS and COVID-19 ARDS subphenotypes using real-world electronic medical record-based data. We validated subphenotypes across the wild-type and delta SARS-CoV2 waves in multiethnic cohorts. We then developed a machine learning subphenotype prediction model with distributable software. We initially hypothesized that we would detect hypo- and hyperinflammatory subphenotypes of hospitalized non-critical COVID-19 patients, like the two subphenotypes identified in non-COVID-19 and COVID-19 ARDS [1,3,4].

## Methods

### Setting, design, and participants

We conducted a retrospective cohort study of patients admitted to New York Presbyterian (NYP) health system hospitals, including Columbia University Medical Center its affiliated community hospital, the Allen Hospital (Columbia), and Cornell Medical Center and its affiliate community hospital, Lower Manhattan Hospital (Cornell). We examined adults age ≥18 years hospitalized from the emergency department (ED) during the peak periods of the wild-type SARS-CoV2 wave (March 6, 2020–June 2, 2020) and delta SARS-CoV2 wave (October 1, 2020–June 2, 2021) in New York City. Study patients had a positive SARS-CoV-2 result on real-time reverse-transcription polymerase chain reaction (PCR) assay from nasopharyngeal swab between 14 days before to 7 days after ED presentation. We excluded those who were intubated or died within 24 hours, those who were discharged alive in < 24 hours, or who had no SpO2 measurement. See S-Methods for additional exclusion criteria. We defined the derivation cohort as Columbia patients from the wild-type wave, the external validation cohort as Cornell patients from the wild-type wave, and the longitudinal validation cohort as Columbia patients from the delta wave. The study was IRB-approved (Columbia-Cornell protocol AAAT3501).

### Data sources

We obtained data from the NYP-Columbia and NYP-Cornell clinical databases (see S-Methods).

### Latent class analysis

For latent class analyses (LCA), we selected demographics, vital signs, and laboratory values that were used in prior non-COVID-19 and COVID-19 ARDS latent class analyses [1,4]. We selected inflammatory markers commonly assessed in clinical care (C-reactive protein [CRP]; ferritin; lactate dehydrogenase [LDH]; erythrocyte sedimentation rate [ESR]), as well as interleukin-6 (IL-6). The same latent class defining variables were used for all three cohorts (S1–S3 Tables), except for IL-6, which was included the LCAs for wild-type and delta wave Columbia cohorts, but not included in the LCAs for the Cornell wild-type cohort since missingness was 91%. We used vital signs ascertained at ED triage and the worst laboratory values within 24 hours of ED presentation. We estimated partial pressure of arterial oxygen to the fraction of inspired oxygen (PaO2:FiO2) from SpO2:FiO2 using validated methods (see S-Methods) [15,16]. In the primary analysis, we used the lowest estimated PaO2:FiO2 in the first 24 hours after presentation rather than the estimated PaO2:FiO2 at ED triage, since supplemental oxygen flow rates were often missing at ED triage for Columbia patients.

To conform to the Gaussian assumptions of LCA, we confirmed that all inflammatory biomarkers had < 15% of values above the upper limit of detection, we log-transformed skewed continuous data, and scaled variables using the z-score. We excluded covariables that were highly correlated (ρ > 0.6). We fit LCA models with 2-5 classes with the full-information maximum likelihood assumption under the missing-at-random assumption. We selected the best fitting model based on the Vuong-Lo-Mendell-Rubin (VLMR) likelihood ratio test, model entropy, Bayesian Information Criteria (BIC), and the size of the smallest class. We assigned each subject to the latent class for which he/she had the maximal probability. In the primary analysis, we excluded those who had no inflammatory plasma biomarkers measured in the first 24 hours because we hypothesized that subphenotypes would be categorized by level of inflammation. We conducted sensitivity LCAs to ensure that subphenotypes were robust to missing data. We repeated LCAs including patients missing all inflammatory biomarkers; excluding variables with > 25% missingness; excluding variables where missingness was associated with death; and using PaO2:FiO2 at ED triage with back fill imputation of FiO2 (Columbia cohorts only). LCA was performed using Mplus8 v1.8.6.

### Characterizing subphenotypes

We compared clinical, biomarker, and outcome variables for subphenotypes using Kruskal-Wallis or χ^2^ tests. We plotted standardized mean values of class-defining variables to visualize similarities between subphenotypes across cohorts. We created Kaplan-Meier plots to assess time from hospital admission to intubation or death with right-censoring at 90 days. We also compared survival between subphenotypes using the restricted mean survival time at 90 days with the *strmst2* command in Stata, which is useful when the proportional hazards assumption may not be met or when the event rate is low [17].

### Subphenotype prediction model

We pooled latent class defining variables (independent variables) and latent class assignment (dependent variable) from the three-class LCA models from each of the three cohorts. We trained XGBoost models to predict subphenotype using 70% of the data (see S-Methods). The model reports the probability of each latent class assignment, assigning the subject to the subphenotype for which he/she has the maximal probability. We tested the final model on 30% of the data and assessed performance via accuracy score (percent when the highest probability predicted latent class is the same as the originally assigned latent class) and area under the receiver-operating-curve (AUC). We calculated variable importance in subphenotype prediction (see S-Methods).

## Results

### Cohort characteristics

There were 2,077 wild-type wave Columbia patients, 1,214 wild-type wave Cornell patients, and 1,536 delta wave Columbia patients included in the primary analyses (S1–S3 Figs in S1 Text). Compared to Cornell patients, more Columbia wild-type and delta wave patients were Hispanic (50% and 53% vs. 25%) or Black (20% and 16% vs. 13%) (Table 1). Cornell wild-type patients had a higher a median Charlson comorbidity score than Columbia wild-type and delta wave patients (2 [1–5] vs. 1 [1–4] and 1 [1–4], respectively). Columbia wild-type patients had lower median nadir PaO2/FiO2 in the first 24 hours after admission than Cornell wild-type and Columbia delta wave patients (150 [64–226] vs. 193 [76–279] and (200 [76–279]) (S4–S6 Tables in S1 Text). Comparing Columbia and Cornell patients during the wild-type wave, intubation rates were lower (14% vs. 20%) and 90-day death rates were higher (23% vs. 12%). Columbia delta wave patients had the longest median time to intubation, lowest intubation rate (4%), and lowest 90-day death rate (15%).

**Table 1:** Characteristics of hospitalized adults with non-critical COVID-19 by cohort and 3-class subphenotype models.

Characteristic	No. avail-able	All	Low-inflammatory	Intermediate-inflammatory	High-inflammatory with fibrinolysis	p-value
**Derivation Cohort - Columbia Wild Type Wave (March 2020-June 2020)**						
No. patients	2077	2077	692	708	677	
Age, years	2077	67 [56–78]	68 [57–79]	58 [47–67]	76 [67–84]	<0.001
Male sex	2077	1190 (57)	313 (45)	471 (67)	406 (60)	<0.001
Body mass index	2077	29 ± 6.8	29 ± 6.7	31 ± 6.8	27 ± 6.2	<0.001
Race	2077					0.262
Black		412 (20)	136 (20)	129 (18)	147 (22)	
White		467 (23)	181 (26)	136 (19)	150 (22)	
Other/Unknown		1198 (58)	375 (54)	443 (63)	380 (56)	
Hispanic Ethnicity	2077	1044 (50)	332 (48)	388 (55)	324 (48)	0.012
Charlson comorbidity index	2077	1 [0–4]	2 [0–4]	0 (0–2)	3 (1–3)	<0.001
SOFA score	1972	3 [2–4]	2 [1–3]	2 [1–3]	4 [3–6]	<0.001
P_a_O_2_/F_i_O_2_ ratio	2055	150 [64.0-226]	229 [168–304]	94.6 [62.5–203]	70.7 [51.0–159]	<0.001
Corticosteroids	2077	561 (27)	91 (13)	239 (32)	240 (34)	<0.001
Tocilizumab	2077	138 (6.6)	9 (1.3)	73 (10)	56 (8.3)	<0.001
Intubation	2077	299 (14)	32 (4.6)	140 (20)	127 (19)	<0.001
Death at 90 days	2077	476 (23)	78 (11)	82 (12)	316 (47)	<0.001
**External Validation Cohort - Cornell Wild Type Wave (March 2020-June 2020)**						
No. patients	1214	1214	630	376	208	
Age, years	1214	67 [56–79]	66 [54–78]	62 [52–71]	82 [70–89]	<0.0001
Male sex	1214	697 (57)	288 (46)	277 (74)	132 (64)	<0.0001
Body mass index	1107	28 ± 7.2	29 ± 7.5	29 ± 6.9	25 ± 5.8	<0.0001
Race	1214					0.0141
Black		157 (13)	93 (15)	35 (9.3)	29 (14)	
White		345 (28)	183 (29)	95 (25)	67 (32)	
Other/Unknown		712 (59)	354 (56)	246 (65)	112 (54)	
Hispanic Ethnicity		299 (25)	178 (28)	79 (21)	42 (20)	0.010
Charlson comorbidity index	1214	2 [1–5]	2 [1–5]	1 [0-3]	5 [2–8]	<0.001
P_a_O_2_/F_i_O_2_ ratio	1214	193 [76–279]	257 [172–319]	94.9 [60.9–198]	101 [64–215]	<0.001
Intubation	1214	247 (20)	69 (11)	124 (33)	54 (26)	<0.001
Death at 90 days	1214	146 (12)	38 (6.0)	27 (7.2)	81 (39)	<0.001
**Longitudinal Validation Cohort - Columbia Delta Wave (October 2020-June 2021)**						
No. patients	1536	1536	541	557	438	
Age, years	1536	67 [54-78]	76 [68-85]	53 [39-62]	71 [61-79]	<0.001
Male sex	1536	785 (51)	264 (49)	288 (52)	233 (53)	0.368
Body mass index	1332	29 ± 6.8	27 ± 6.1	32 ± 7.0	28 ± 6.2	<0.001
Race	1536					0.091
Black		243 (16)	98 (18)	82 (15)	63 (14)	
White		391 (26)	149 (28)	142 (26)	100 (23)	
Other/Unknown		902 (59)	294 (54)	333 (60)	275 (63)	
Hispanic Ethnicity	1536	819 (53)	273 (51)	318 (57)	228 (52)	0.073
Charlson comorbidity index	1536	1 [0–4]	3 [1–6]	0 [1,2]	1 [0-4]	<0.001
SOFA score	1302	2 [1–3]	2 [1–4]	2 [1–4]	3 [2–4]	<0.001
P_a_O_2_/F_i_O_2_ ratio	1536	200 [76–269]	236 [171–304]	217 [134–280]	76.0 [47–189]	<0.001
Corticosteroids	1536	1218 (79)	346 (48)	471 (85)	401 (92)	<0.001
Tocilizumab	1536	37 (2.4)	3 (0.5)	15 (2.6)	19 (4.3)	0.001
Intubation	1536	64 (4.2)	12 (2.2)	21 (3.8)	31 (7.1)	<0.001
Death at 90 days	1536	224 (15)	70 (13)	25 (4.5)	129 (30)	<0.001

SOFA from time of ED admission was not available in the Cornell CEDAR database. See S12–S14 Tables for comorbidity, physiologic, and laboratory data for each of the three subphenotypes in each of the three cohorts.

### Latent class analyses

**Fig 1:**
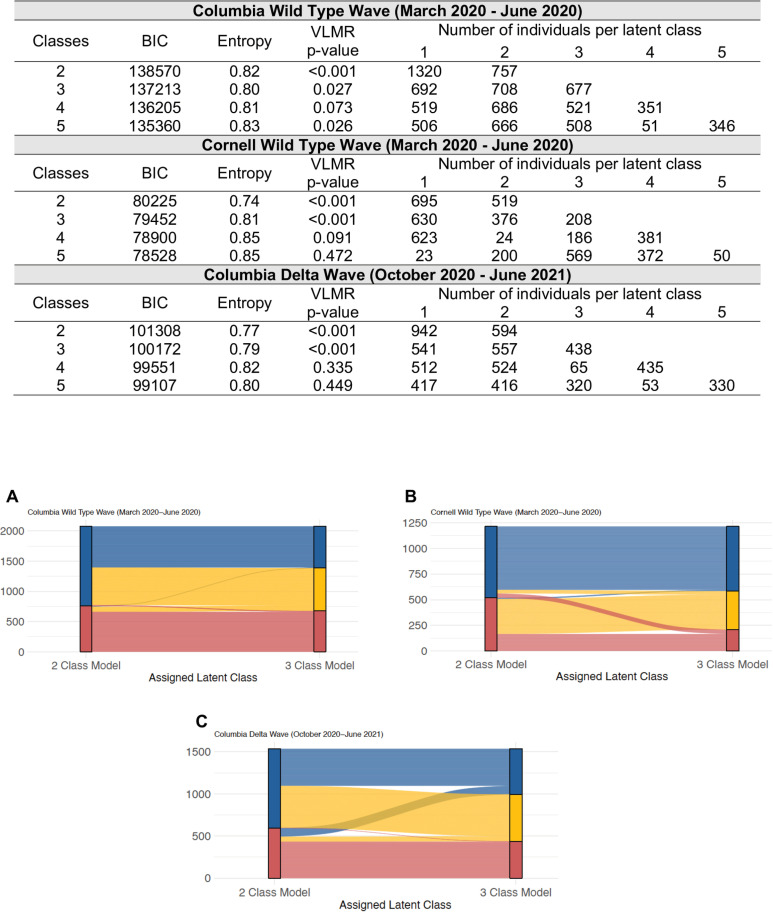
Latent class model fit statistics and alluvial diagrams showing patient-latent class transitions between two and three class models. **(A)** Columbia wild-type wave (derivation) cohort, **(B)** Cornell wild-type wave (external validation) cohort, and **(C)** Columbia delta wave (longitudinal validation) cohort. BIC: Bayesian Information Criterion. Entropy is a measure of latent class separation. VLMR: Vuong-Lo-Mendell-Rubin likelihood ratio tests whether the number of classes provides improved model fit with a model using one fewer class. See S7 Table in S1 Text for average latent class membership probabilities.

Across the three cohorts, the VLMR test consistently showed that two-class and three-class models were significant improvements over models with one fewer class (Fig 1). Entropy was nearly 0.80 or higher for two- and three-class models in all cohorts, indicating good separation of classes. Compared to the two-class models in the Cornell wild-type and Columbia delta wave cohorts, entropy was higher for the three-class models. The BIC decreased between all 2- and 3-class models. Class size became small in 4- and 5-class models. Mean probabilities for class membership in the 3-class models were all ≥ 0.90 (S7 Table in S1 Text). Across the cohorts, patients from a single class in the two-class models were primarily split into two classes in the three-class models (Fig 1A–C).

A total of 332 (14%), 239 (17%), and 558 (27%) patients from the Columbia wild-type, Cornell wild-type, and Columbia delta wave cohorts were excluded in primary analyses due to lack of inflammatory plasma biomarker measurements during the first 24 hours after admission (S1–S3 Figs in S1 Text). Excluded patients had higher PaO2/FiO2 ratios and lower intubation and death rates (S4–S6 Tables). Two- and three-class models across the cohorts were robust to sensitivity analyses including these patients without inflammatory biomarkers, with lower BIC levels favoring the three-class models (S8 Table in S1 Text). Two- and three-class models were also robust to sensitivity analyses excluding variables with > 25% missingness (S1–S3 and S9 Tables in S1 Text), and excluding variables that were possibly missing not-at-random due to death (S1–S3 and S10 Tables in S1 Text), except the third class size became small in the Cornell wild-type cohort when troponin was excluded as a class-defining variable. Two- and three-class models in the Columbia cohorts were robust to PaO2:FiO2 at ED triage with back fill imputation of FiO2 (S11 Table in S1 Text). Given the robustness of the two- and three-class models and lower BIC and higher entropy for the 3-class models, we examined characteristics and outcomes for both two- and three-class models but retained the three-class models for primary analyses and subphenotype prediction modeling.

### Subphenotype characteristics

**Fig 2:**
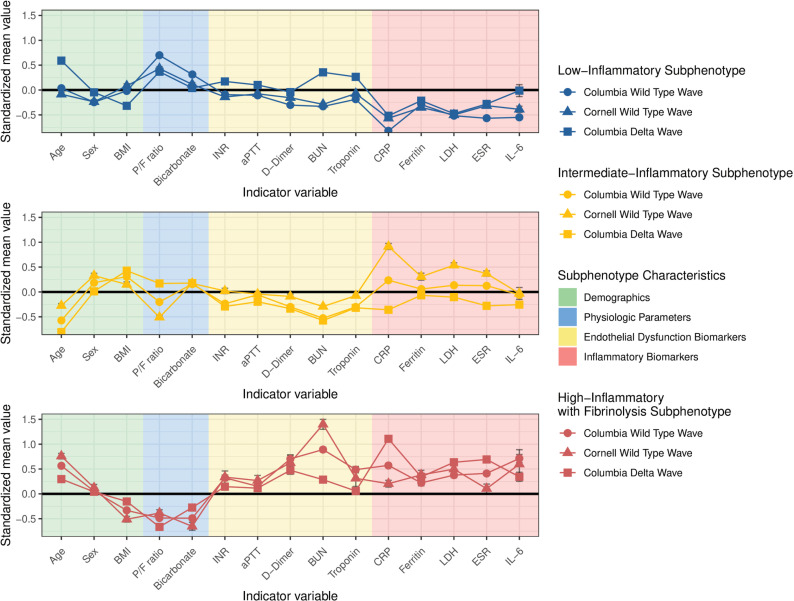
Comparison of differences in standardized values of variables for each of the three subphenotypes across the Columbia wild-type, Cornell wild-type, and Columbia delta wave cohorts. Variables were standardized to a z score for each cohort where the means were scaled to zero and the standard deviations to one. BMI: body mass index. P/F ratio: partial pressure of oxygen/fraction of inspired oxygen ratio. INR: international normalization ratio. aPTT: activated partial thromboplastin time. BUN: blood urea nitrogen. CRP: C-reactive protein. LDH: lactate dehydrogenase. ESR: Erythrocyte sedimentation rate. IL-6: interleukin-6.

**Fig 3:**
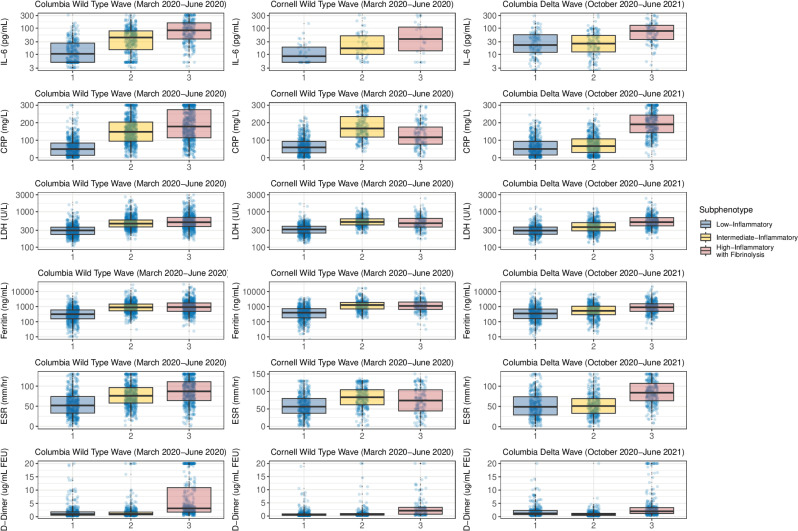
Dot-box plots of plasma levels of inflammatory biomarkers and D-dimer. Boxes represent the interquartile range, and the middle bar represents the median. *P* values for each plot are all < 0.001. The upper limit of detection for D-Dimer assay is 20 ug/ml FEU at Columbia and 200 ug/ml at Cornell. To plot Columbia and Cornell data on the same axes, Cornell patient values of > 20 ug/ml were assigned a level of 20 ug/ml. Median [IQR] values of each of the biomarkers are listed in S12–S14 Tables in S1 Text. IL-6: interleukin-6. CRP: C-reactive protein. LDH: lactate dehydrogenase. ESR: Erythrocyte sedimentation rate.

Several endothelial and inflammatory biomarker levels were similar among subphenotypes across the cohorts suggesting that subphenotypes were pathobiologically robust in a diverse population spanning the wild-type and delta SARS-Cov2 waves (Fig 2). IL-6 increased across classes 1-3 in all cohorts. In the Columbia wild-type and delta wave cohorts, CRP, LDH, ferritin, and ESR generally increased across classes 1-3, but in the Cornell wild-type cohort, LDH, ferritin, and ESR were similar in the classes 2 and 3, and CRP was lower in class 3 than in class 2 (Fig 3). Class 3 had the highest standard mean D-Dimer across all cohorts. Class 3 had the highest BUN and troponin levels in the wild-type wave cohorts, but BUN and troponin levels were similar to those of class 1 in the delta wave (Fig 2 and S12–S14 Tables in S1 Text). Since we consistently observed in all cohorts a pattern of increasing inflammation across the three classes and fibrinolysis in class 3, we named class 1 low-inflammatory, class 2 intermediate-inflammatory, and class 3 high-inflammatory with fibrinolysis.

The intermediate-inflammatory subphenotype had the youngest median age and the lowest Charlson comorbidity index in all cohorts. The highest median age and comorbidity burden changed from the high-inflammatory subphenotype in the Columbia and Cornell wild-type cohorts, to the low-inflammatory subphenotype in the Columbia delta wave cohort (Table 1).

Vital signs did not appear to be clinically different between subphenotypes, though they were sometimes statistically significantly different (S12–S14 Tables in S1 Text). The low-inflammatory subphenotype had the highest median PaO2/FiO2 ratio, ranging from 229-257 across the three cohorts (Table 1). In the Columbia and Cornell wild-type cohorts, the intermediate- and high-inflammatory subphenotypes had the lowest median PaO2/FiO2 ranging from 71-101. However, in the Columbia delta wave cohort, the intermediate-inflammatory subphenotype had a comparably higher median PaO2/FiO2 of 217 [134-280], while the high-inflammatory with fibrinolysis subphenotype continued to have a low median PaO2/FiO2 of 76 [47-189].

Comparing the Columbia wild-type to the delta wave cohort, corticosteroid use in the low-, intermediate-, and high-inflammatory subphenotypes increased from 13% to 48%, 32% to 85%, and 34% to 92%, respectively.

**Fig 4:**
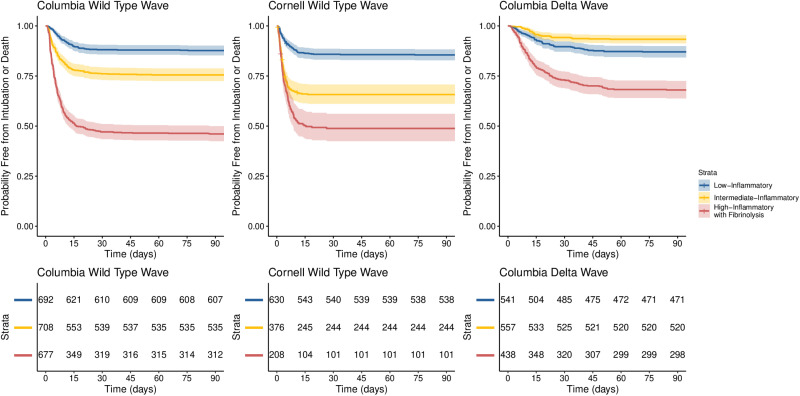
Kaplan-Meier plots of time to intubation or death right-censored at 90 days. Plots are stratified by the 3-class latent class model of COVID-19 in the Columbia wild-type wave (derivation cohort), the Cornell wild-type wave (external validation cohort), and the Columbia delta wave (longitudinal validation cohort). All log-rank ****p**** < 0.001.

In the Columbia and Cornell wild-type wave cohorts, the low-, intermediate-, and high-inflammatory subphenotypes had 90-day intubation or death rates of 12% and 15%, 24% and 35%, and 54% and 51%, respectively (Fig 4 and S12–S14 Tables in S1 Text). However, in the Columbia delta wave cohort, the intermediate-inflammatory subphenotype had the lowest 90-day intubation or death rate of 6.6%, whereas the low- and high-inflammatory subphenotypes had 90-day intubation or death rates of 13% and 32%, respectively (all *p* < 0.001). Similar statistically significant relationships were observed in restricted mean survival time analyses (S15 Table in S1 Text).

The two-class models revealed hypo- and hyperinflammatory subtypes, with the hyperinflammatory subphenotype being older, with greater comorbidity, lower PaO2/FiO2 ratio, and higher 90-day intubation or death rates (S16–S18 Tables and S4 and S5 Figs in S1 Text). The two-class hypoinflammatory subphenotype had greater median levels of inflammatory biomarkers than the 3-class low-inflammatory subtype. D-Dimer levels were consistently greater in the hyperinflammatory subphenotype across all 3 cohorts (all *p* < 0.001).

### Subphenotype prediction model performance

**Fig 5:**
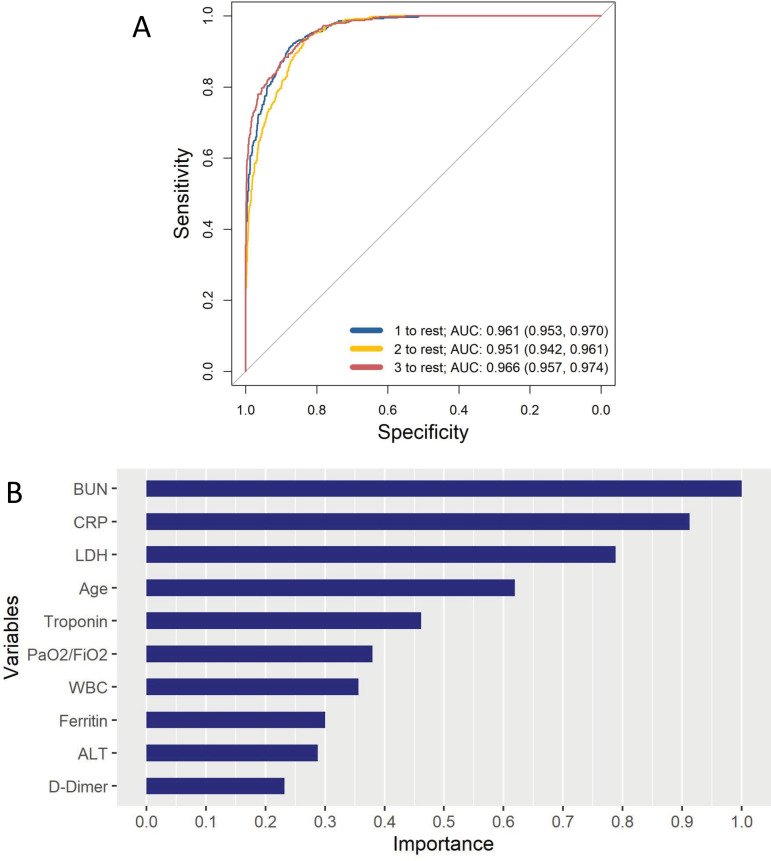
XG Boost subphenotype model characteristics. **(A)** Receiver Operating Characteristic curves for predicting each of the 3 subphenotypes in the testing dataset. **(B)** Top 10 most important partitioning variables in the XGBoost model. AUC: area under the receiver operating characteristics curve. BUN: blood urea nitrogen. CRP: C-reactive protein. LDH: lactate dehydrogenase. PaO2/FiO2: partial pressure of oxygen to fraction of inspired oxygen ratio. WBC: white blood cell count, ALT: alanine aminotransferase.

The XGBoost subphenotype prediction model had an AUC of 0.96 (95%CI 0.95-0.97) for subphenotype 1, 0.95 (95%CI 0.94-0.96) for subphenotype 2, and 0.97 (0.96-0.97) for subphenotype 3, with an overall average AUC of 0.96 in the testing dataset (Fig 5A). Model accuracy score on the testing data was 83%. Model performance was robust when the seed parameter was changed four times (S19 Table) in S1 Text. We created a model without IL-6, since it is often not measured. The model had an identical average AUC of 0.96 and accuracy of 83%. R compatible software and instructions for using the prediction model can be downloaded from the online supplement.

The top 10 most important variables in the model are presented in Fig 5B. BUN is the strongest predictor in the model, with troponin being another strong extrapulmonary biomarker predictor (Fig 5B). CRP is the strongest inflammatory biomarker predictor, with LDH and ferritin being other important inflammatory biomarker predictors. PaO2/FiO2 is the sixth most important predictor. Vital signs, sex, and BMI, which are important predictors of outcomes in COVID-19, were not prominent latent class partitioning variables.

## Discussion

In large New York City-based multiethnic cohorts spanning the wild-type and delta SARS-CoV2 waves, we consistently identified three subphenotypes of adults hospitalized with non-critical COVID-19. Subphenotypes were characterized by low-inflammation, intermediate-inflammation, and high-inflammation with fibrinolysis that were robust to changes in age and comorbidity burden over time, and that did not just reflect increasing levels COVID-19 severity since subphenotypes had similar vital signs and a wide distribution of PaO2/FiO2 ratios. The marked relative improvement in the 90-day intubation or death rate in the intermediate-inflammatory subphenotype from the wild-type to the delta wave suggests that this group may have benefitted most from the implementation of standardized corticosteroid therapy, and suggests the possibility of heterogeneity-of-treatment effect in other drugs tested to prevent progression of COVID-19. Our machine learning model software may facilitate post-hoc heterogeneity-of-treatment effect analyses of trials with hospitalized non-critical COVID-19 patients that have found mostly null average treatment effects.

Our findings suggest that the hypo- and hyper-inflammatory subphenotypes in intubated COVID-19 ARDS patients emerge prior to the onset of critical illness [18]. While the LCAs suggested that the data best fit a three-class model, there was strong evidence for a two-class model with hypo- and hyper-inflammatory characteristics. In the three-class model, the intermediate-inflammatory and high-inflammatory with fibrinolysis COVID-19 subphenotypes had substantial intubation and death rates in the wild-type wave with many patients appearing like predecessors of the hypoinflammatory low fibrinolysis and hyperinflammatory high-fibrinolysis subphenotypes of intubated COVID-19 ARDS patients that we identified in our prior work [18]. From the wild-type to the delta wave, we observed that the oldest patients with the highest comorbidity burden shifted from the high-inflammation with fibrinolysis subphenotype to the low-inflammation subphenotype. This may reflect the fact that older adults and those with comorbid conditions were offered access to SARS-CoV2 vaccination first, and suggests that we identified pathobiologically distinct subphenotypes robust to changing demographics and comorbidities.

There are well-validated COVID-19 prediction models for clinical decompensation and mortality [19,20], and prior studies identified non-critical COVID-19 subphenotypes via cluster modeling [10,11,14]. However, these studies included only wild-type wave patients. Our XGBoost model is an advancement in COVID-19 prediction modeling because it incorporates both wild-type and delta wave patients, allows for partial missing data, consists of variables regularly measured in clinical care, identifies pathobiologically distinct subphenotypes with different risks of intubation and death, and has distributable software.

It is biologically plausible that the subphenotypes we identified could reveal heterogeneity-of-treatment effect in post-hoc analyses of trials with hospitalized non-critical COVID-19 patients. In the ACTIV-1 trial, abatacept and infliximab did not improve time to recovery but reduced 28-day mortality [21]. In the ACTIV-3a TICO trial, Tixagevimab-cigavimab similarly did not improve time to recovery but significantly reduced 28-day mortality [22]. In the Recovery trial, with baricitinib reduced 28-day mortality by only 2% [23]. Analyses stratified by the subphenotypes that we identified could plausibly reveal greater benefit in the intermediate or high-inflammation subphenotypes due to predictive or prognostic enrichment [24]. Indeed, a post-hoc cluster analysis of hospitalized COVID-19 patients in an imatinib trial suggested that a subgroup with high inflammation and endothelial dysfunction benefitted most [25]. In the ACT trial, aspirin and rivaroxaban did not improve outcomes [26]. In the Recovery trial, aspirin did not reduce 28-day mortality, but was associated with an increased rate of being discharged alive by 28 days [27]. Analyses stratified by the high-inflammation with fibrinolysis subphenotype could plausibly reveal a benefit for the anti-thrombotic effects of aspirin and rivaroxaban in this subphenotype with dysregulated coagulation.

Our study has limitations. Our cohort is racially and ethnically diverse, but we sampled only from New York City. We did not include patients from the omicron wave because a high proportion were asymptomatic [28]. Our nadir SpO2/FiO2 may underestimate the true PaO2/FiO2 if a low SpO2 was due to measurement artifact. The third-class size became small in the Cornell cohort in a sensitivity analysis excluding troponin due to possible missingness associated with death, but split gain analyses revealed that troponin is an important subphenotype partitioning variable. We ascertained death dates from the Epic Systems electronic medical record (Verona, Wisconsin), as state and federal death index databases for 2020 and 2021 are not yet available [29].

## Conclusion

In large multiethnic cohorts we consistently identified subphenotypes of low-inflammation, medium-inflammation, and high-inflammation with fibrinolysis in hospitalized adults with non-critical COVID-19 using real-world electronic medical record data that were robust to temporal changes in demographics and comorbidities. Post-hoc analyses of trials from prior waves using our prediction model may reveal heterogeneity-of-treatment effects that could inform patient care should a more virulent SARS-CoV-2 strain arise in the future. Our findings also provide proof-of-concept that similar non-critical subphenotypes may be identifiable in other viral and bacterial pneumonias, which in turn, could lead to a new era of predictively and prognostically enriched immunomodulator trials to prevent progression of non-critical ALI to ARDS.

## Supporting information

S1 Text Supporting figures and tables.
(PDF)
