# Supplementary material for: Three hospitalized non-critical COVID-19 subphenotypes and change in intubation or death over time: A latent class analysis with external and longitudinal validation
Source: PLoS One. 2025 Mar 19;20(3):e0316434. doi: 10.1371/journal.pone.0316434 (PMC11922525; doi:10.1371/journal.pone.0316434)
Supplement: S1 Text — (PDF) [file pone.0316434.s001.pdf]

Three Hospitalized Non-Critical COVID-19 Subphenotypes and Change in Intubation or Death Over Time

William S. Stringer, MD; Amy S. Labar, MD, MS; Joshua D. Geleris, MD; Evan V. Sholle, MS; David A. Berlin, MD; Claire M. McGroder, MD, MS; Matthew J. Cummings, MD, MS Max R. O'Donnell, MD, MPH;[1] Haoyang Yi, MS; Xuehan Yang, BS; Ying Wei, PhD;[4] Edward J. Schenck, MD, MS; Matthew R. Baldwin, MD, MS

ONLINE SUPPORTING INFORMATION

SUPPLEMENT TABLE OF CONTENTS

S-Methods……………………………………………………..3
S1 Table…………………………………………….......4
S2 Table…………………………………………….......5
S3 Table……………………………………………………...6
S4 Table……………………………………………………...7
S5 Table……………………………………………………...9
S6 Table……………………………………………………...11
S7 Table……………………………………………………...13
S8 Table……………………………………………………...14
S9 Table……………………………………………………...15
S10 Table……………………………………………………16
S11 Table……………………………………………………17
S12Table……………………………………………………18
S13 Table……………………………………………………20
S14 Table……………………………………………………22
S15 Table……………………………………………………24
S16 Table……………………………………………………26
S17 Table……………………………………………………28
S18 Table……………………………………………………30
S1 Figure……………………………………………………..31
S2 Figure……………………………………………………..32
S3 Figure……………………………………………………..33
S4 Figure……………………………………………………..34
S5 Figure……………………………………………………..35
S References……………………………………………..36

S-Methods

Participant exclusion criteria

We reviewed patients with SARS-CoV2 positive nasal swabs 7-14 days prior or 3-7 days after admission, excluding those hospitalized for reasons unrelated to COVID-19 or who possibly had nosocomial SARS-CoV-2 infection, respectively. We reviewed patients with white blood cell counts and total bilirubin levels that were greater than each study cohort's 99th percentile and excluded patients without respiratory symptoms or hypoxemia who were admitted for extrapulmonary bacterial infections or acute decompensated cirrhosis, respectively.

Data sources

The Columbia clinical data warehouse comprises over 30 years of data on over six million patients from the New York-Presbyterian (NYP) / Columbia University Irving Medical Center (CUIMC), collected from electronic health records over time, currently from Epic Systems (Verona, Wisconsin).[1] The data include all outpatient and inpatient demographic characteristics, visit information, diagnoses, procedures, including intubation and mechanical ventilation use, medications, vital signs, care provider notes, orders and prescriptions, laboratory results, radiology reports, and numerous other ancillary reports. Laboratory and ancillary data are fed directly to the warehouse from the source computing systems and serve as the gold standard for data quality reviews for clinical trials. The Observational Health Data Sciences and Informatics initiative data quality tool set called Achilles Heel includes an extensive knowledge base of data consistency checks used to verify the quality of the Columbia warehouse.[2]

The NYP-Cornell Critical care Database for Advanced Research (CEDAR) automatically extracts and transforms data from Cornell-based electronic health record systems on over 550 variables, and has been shown to be accurate and comparable with manual data extraction. In spring 2020, the CEDAR data extraction algorithm was modified to support clinical operations and research during the SARS-CoV2 pandemic in New York City.[3,4]

For all cohorts, we assessed comorbid conditions based on groups of ICD-10 diagnosis codes according to the Clinical Classifications Software by the Healthcare Cost and Utilization Project[5] and according to an ICD-10 version of the Charlson comorbidity index.[6] The PaO2/FiO2 ratio was calculated by estimating the PaO2 from the lowest recorded oxygen saturation recorded in the first 24 hours after emergency department presentation, and then dividing by the estimated FiO2 for that oxygen delivery method (0.21 for room air, 0.21 ± (oxygen flow rate x 0.03) for nasal cannula, 0.80 for non-rebreather mask,[7,8] or the recorded FiO2 for non-invasive positive pressure ventilation or high flow nasal cannula oxygen, as we have done in our prior COVID-19 studies.[9,10]

We ascertained death dates from NYP-CUIMC clinical data warehouse, NYP-CEDAR database, and Epic Systems electronic medical record (Verona, Wisconsin), which includes deaths registered from any medical facility that uses EPIC software. City, state, and federal death index databases for 2020 and 2021 were not yet available at the time of analysis.[11]

XGBoost methods

XGBoost uses successive decision trees to attempt to correct the classification error of previous trees in the model, allowing for iterative refinement of the model and maximally optimized classification. We trained the XGBoost model to predict subphenotype using minimization of multiclass classification error rate as the model's learning objective. To tune hyperparameters, we performed 5-fold cross validation using a grid search protocol with 3 hyperparameters of interest (max depth, number of rounds ensembled, and learning rate). We repeated this four more times with different seeds to evaluate model robustness to seed sensitivity (Table E18). We conducted 45 rounds of cross validation and used the maximal accuracy on the out of bag fold metric to identify the ideal set of hyperparameters. We calculated variable importance in subphenotype prediction using the split gain for each variable across the different trees in the models. Analyses were conducted using the XGBoost R package in RStudio (version 3.6.2).

SUPPORTING INFORMATION RESULTS

S1 Table. Latent Class Defining variables, missingness, and associations of missingness with death for the derivation cohort (Columbia wild type wave cohort).

Variable	n (%) Missing, All	n (%) Missing, Died	n (%) Missing, Survived	p-value
Age (years)	0 (0)	0 (0)	0 (0)	N/A
Sex	0 (0)	0 (0)	0 (0)	N/A
**Body Mass Index (kg/m^2))**	**368 (18)**	**141 (30)**	**227 (14)**	**<0.01**
Temperature (^oC)	1 (0.1)	1 (0.2)	0 (0)	0.07
Heart Rate (beats/min)	2 (0.1)	0 (0)	2 (0.1)	0.44
Respiratory Rate (per min)	4 (0.2)	1 (0.2)	3 (0.2)	0.92
PaO_2/FiO_2	0 (0)	0 (0)	0 (0)	N/A
Mean Arterial Pressure (mmHg)	1 (0.1)	0 (0)	1 (0.1)	0.59
Troponin (ng/L)	252 (12)	60 (13)	192 (12)	0.72
International Normalization Ratio	399 (19)	86 (18)	313 (20)	0.47
Activated Partial Thromboplastin Time (seconds)	412 (20)	83 (17)	329 (21)	0.14
**D-Dimer (mcg/mL)**	**809 (39)**	**223 (47)**	**586 (37)**	**<0.01**
White Blood Cells ($x10^{-9}/L$)	34 (1.6)	11 (2.3)	23 (1.4)	0.19
Lymphocyte Count ($x10^{-9}/L$)	42 (2.0)	16 (3.4)	26 (1.6)	0.02
Hemoglobin (g/dL)	17 (0.8)	5 (1.1)	12 (0.8)	0.52
Platelets ($x10^{-9}/L$)	28 (1.4)	8 (1.7)	20 (1.3)	0.47
Blood Urea Nitrogen (mg/dL)	11 (0.5)	2 (0.4)	9 (0.6)	0.71
Bicarbonate (mmol/L)	12 (0.6)	3 (0.6)	9 (0.6)	0.86
Sodium (mmol/L)	11 (0.5)	2 (0.4)	9 (0.6)	0.71
Bilirubin total (mg/dL)	84 (4.0)	26 (5.5)	58 (3.6)	0.07
Alanine Aminotransferase (U/L)	95 (4.6)	30 (6.3)	65 (4.1)	0.04
Albumin (g/dL)	77 (3.7)	22 (4.6)	55 (3.4)	0.23
C-Reactive Protein (mg/L)	177 (8.5)	36 (7.6)	141 (8.8)	0.39
Ferritin (mg/dl)	251 (12)	60 (13)	191 (12)	0.69
**Interleukin-6 (pg/ml)**	**1170 (56)**	**302 (64)**	**868 (54)**	**<0.01**
Lactate Dehydrogenase (U/L)	211 (10)	49 (10)	162 (10)	0.91
Erythrocyte Sedimentation Rate (mm/hr)	483 (23)	106 (22)	377 (24)	0.56

Bolded variables are excluded in sensitivity analyses due to >25% missing or missingness being associated with death.

S2 Table. Latent Class Defining variables, missingness, and associations of missingness with death for the external validation cohort (Cornell wild type wave cohort).

Variable	n (%) Missing, All	n (%) Missing, Died	n (%) Missing, Survived	p-value
Age (years)	0 (0)	0 (0)	0 (0)	N/A
Sex	0 (0)	0 (0)	0 (0)	N/A
Body Mass Index (kg/m^2))	107 (8.8)	16 (11)	91 (8.5)	0.33
Temperature (^{o}C)	0 (0)	0 (0)	0 (0)	N/A
Heart Rate (beats/min)	0 (0)	0 (0)	0 (0)	N/A
Respiratory Rate (per min)	0 (0)	0 (0)	0 (0)	N/A
PaO_2/FiO_2	0 (0)	0 (0)	0 (0)	N/A
Mean Arterial Pressure (mmHg)	0 (0)	0 (0)	0 (0)	N/A
**Troponin (ng/L)**	**167 (14)**	**10 (6.9)**	**157 (15)**	**0.01**
International Normalization Ratio	183 (15)	16 (11)	167 (16)	0.14
Activated Partial Thromboplastin Time (seconds)	218 (18)	18 (12)	200 (19)	0.06
**D-Dimer (mcg/mL)**	**393 (32)**	**53 (36.3)**	**340 (32)**	**0.28**
White Blood Cells (x10^{-9}/L)	14 (1.2)	3 (2.1)	11 (1.0)	0.28
Lymphocyte Count (x10^{-9}/L)	29 (2.4)	3 (2.1)	26 (2.4)	0.78
Hemoglobin (g/dL)	0 (0)	0 (0)	0 (0)	N/A
Platelets (x10^{-9}/L)	3 (0.3)	1 (0.7)	2 (0.2)	0.26
Blood Urea Nitrogen (mg/dL)	2 (0.2)	0 (0)	2 (0.2)	0.60
Bicarbonate (mmol/L)	2 (0.2)	0 (0)	2 (0.2)	0.60
Sodium (mmol/L)	2 (0.2)	0 (0)	2 (0.2)	0.60
Bilirubin total (mg/dL)	22 (1.8)	1 (0.7)	21 (2.0)	0.28
Alanine Aminotransferase (U/L)	22 (1.8)	1 (0.7)	21 (2.0)	0.28
Albumin (g/dL)	22 (1.8)	1 (0.7)	21 (2.0)	0.28
C-Reactive Protein (mg/L)	299 (24.6)	43 (30)	256 (24)	0.15
Ferritin (mg/dl)	205 (17)	23 (16)	256 (17)	0.70
Interleukin-6 (pg/ml)*	1099 (91)	133 (91)	966 (91)	0.80
Lactate Dehydrogenase (U/L)	106 (8.7)	18 (12)	88 (8.2)	0.10
**Erythrocyte Sedimentation Rate (mm/hr)**	**374 (31)**	**46 (32)**	**328 (31)**	**0.85**

*Since Interleukin-6 (IL-6) missingness was 91%, IL-6 was not included as a class defining variable in the Cornell Wild Type wave latent class analyses. Bolded variables are excluded in sensitivity analyses due to >25% missing or missingness being associated with death.

S3 Table. Latent Class Defining variables, missingness, and associations of missingness with death for the longitudinal validation cohort (Columbia delta wave cohort).

Variable	n (%) Missing, All	n (%) Missing, Died	n (%) Missing, Survived	p-value
Age (years)	0 (0)	0 (0)	0 (0)	N/A
Sex	0 (0)	0 (0)	0 (0)	N/A
**Body Mass Index (kg/m^2)**	**204 (13)**	**65 (29)**	**139 (11)**	**<0.001**
Temperature (^{o}C)	3 (0.2)	2 (0.9)	1 (0.1)	0.011
Heart Rate (beats/min)	1 (0.1)	0 (0)	1 (0.1)	0.68
Respiratory Rate (per min)	0 (0)	0 (0)	0 (0)	N/A
PaO_2/FiO_2	0 (0)	0 (0)	0 (0)	N/A
Mean Arterial Pressure (mmHg)	3 (0.2)	0 (0)	3 (0.2)	0.47
**Troponin (ng/L)**	**263 (17)**	**16 (7.1)**	**247 (19)**	**<0.001**
**International Normalization Ratio**	**356 (23)**	**31 (13)**	**325 (25)**	**<0.001**
**Activated Partial Thromboplastin Time (seconds)**	**360 (23)**	**37 (16)**	**323 (25)**	**0.008**
**D-Dimer (mcg/mL)**	**438 (29)**	56 (25)	382 (29)	0.207
White Blood Cells (x10^{-9}/L)	16 (1.0)	2 (0.9)	14 (1.0)	0.81
Lymphocyte Count (x10^{-9}/L)	69 (4.5)	9 (4.0)	60 (4.6)	0.711
Hemoglobin (g/dL)	3 (0.2)	0 (0)	3 (0.2)	0.47
Platelets (x10^{-9}/L)	15 (1.0)	3 (1.3)	12 (0.9)	0.55
Blood Urea Nitrogen (mg/dL)	5 (0.3)	0 (0)	5 (0.4)	0.36
Bicarbonate (mmol/L)	4 (0.3)	0 (0)	4 (0.3)	0.41
Sodium (mmol/L)	3 (0.2)	0 (0)	3 (0.2)	0.47
Bilirubin total (mg/dL)	55 (3.6)	6 (2.7)	49 (3.7)	0.43
Alanine Aminotransferase (U/L)	61 (4.0)	7 (3.0)	54 (4.1)	0.48
Albumin (g/dL)	54 (3.5)	6 (2.7)	48 (3.7)	0.46
C-Reactive Protein (mg/L)	164 (11)	23 (10)	141 (11)	0.83
Ferritin (mg/dl)	329 (21)	43 (19)	286 (22)	0.38
**Interleukin-6 (pg/ml)***	**1139 (74)**	167 (75)	972 (74)	0.88
Lactate Dehydrogenase (U/L)	**388 (26)**	51 (23)	337 (26)	0.35
**Erythrocyte Sedimentation Rate (mm/hr)**	**575 (37)**	**80 (36)**	**495 (38)**	**0.56**

Bolded variables are excluded in sensitivity analyses due to >25% missing or missingness being associated with death.

S4 Table. Comparison of primary analysis patients with patients excluded for having all inflammatory markers missing in the derivation cohort (Columbia wild type wave).

	No. available	All	Included: 1-5 inflammatory biomarkers	Excluded: inflammatory markers missing	p-value
**No. patients**	2410	2410	2077	333	
**Demographics**					
Age, years	2410	67 [55-78]	67 [56-78]	67 [54-79]	0.836
Male sex	2410	1368 (57)	1190 (57)	178 (54)	0.210
Body mass index	1992	29 ± 6.8	29 ± 6.8	28 ± 6.7	0.263
Race	2410				
Black		491 (20)	412 (20)	79 (24)	0.118
White		538 (22)	467 (23)	71 (21)	0.688
Other/Unknown		1381 (57)	1198 (58)	183 (55)	0.382
Hispanic	2410	1224 (51)	1044 (50)	180 (54)	0.221
**Comorbidities**					
Any Below Comorbidity	2410	1600 (66)	1384 (67)	216 (64)	0.567
Cancer	2410	399 (17)	354 (17)	45 (14)	0.126
Chronic kidney disease	2410	502 (21)	435 (21)	67 (20)	0.787
Chronic heart failure	2410	420 (17)	355 (17)	65 (20)	0.314
Diabetes (without complications)	2410	945 (39)	824 (40)	121 (36)	0.273
Diabetes (with complications)	2410	572 (24)	485 (23)	87 (26)	0.300
Essential hypertension	2410	1321 (55)	1141 (54)	180 (54)	0.810
Smoker (current or former)	2410	304 (13)	259 (12)	45 (14)	0.657
Charlson comorbidity index	2410	1 [0-4]	1 [0-4]	1 [0-4]	0.183
**Laboratory**					
Albumin, g/dL	2257	3.7 ± 0.5	3.7 ± 0.5	3.9 ± 0.6	<0.001
Alanine aminotransferase, U/L	2236	28 [18-49]	29 [18-50]	24 [15-43]	<0.001
Aspartate aminotransferase, U/L	2249	42 [27-68]	43 [28-70]	36 [22-55]	<0.001
Bilirubin, mg/dL	2250	0.5 [0.3-0.7]	0.5 [0.3-0.7]	0.4 [0.3-0.7]	0.192
Hemoglobin, g/dL	2381	12.9 ± 2.3	12.9 ± 2.3	12.8 ± 2.3	0.357
WBC, x10^9/L	2364	7.3 [5.5-10]	7.3 [5.5-10]	7.5 [5.5-9.8]	0.854
Percent Lymphocytes	2351	13 [8.9-20]	13 [8.6-20]	16 [11-25]	<0.001
Percent Neutrophils	2281	75 [63-83]	75 [64-83]	72 [60-80]	0.001
Neutrophil-Lymphocyte Ratio	2266	4.8 [2.6-8.5]	5.0 [2.7-7.2]	4.0 [2.2-7.2]	<0.001
Platelets, x10^9/L	2368	204 [154-264]	203 [155-265]	204 [151-257]	0.348
D-dimer, ug/mL FEU	1285	1.36 [0.77-2.79]	1.37 [0.77-2.81]	0.77 [0.45-1.47]	0.030
International Normalized Ratio	1802	1.1 [1.0-1.2]	1.1 [1.0-1.2]	1.1 [1.1-1.2]	0.017
aPTT, second(s)	1791	33 [30-37]	33 [30-37]	32 [29-36]	0.085
BUN, mg/dL	2385	19 [14-34]	19 [12-35]	19 [12-33]	0.560
Creatinine, mg/dL	2385	1.08 [0.82-1.66]	1.08 [0.82-1.67]	1.1 [0.81-1.65]	0.964
Bicarbonate, mmol/L	2384	23 [20-25]	22 [20-25]	23 [20-26]	0.009

Sodium, mmol/L	2385	138 [134-141]	137 [134-140]	138 [135-141]	0.004
Troponin-T, ng/L	2006	17 [8-41]	17 [8-40]	24 [11-46]	0.013
**Vitals**					
Mean Arterial Pressure, mmHg	2409	93 [83-102]	92 [82-101]	94 [83-105]	0.062
Temperature, ˚F	2409	99.3 ± 1.6	99.3 ± 1.6	98.9 ± 1.5	<0.001
Heart Rate, /min	2407	97 ± 19	98 ± 19	94 ± 19	<0.001
P_aO_2/F_iO_2 ratio	2383	164 [66-248]	150 [64-226]	263 [131-323]	<0.001
Respiratory Rate, per min	2406	20 [18-22]	20 [18-22[	18 [18-20]	<0.001
SOFA score	2225	2 [1-4]	3 [2-3]	2 [1-3]	<0.001
**Outcomes**					
Intubation	2410	322 (13)	299 (14)	23 (6.9)	<0.001
Death at 90 days	2410	534 (22)	476 (23)	58 (17)	0.030
Intubation or Death	2410	689 (29)	623 (30)	66 (20)	<0.001
DNR history	2410	130 (5.3)	103 (4.9)	27 (8.1)	0.026
Time to Intubation, hours	322	88.8 [45.1-181]	87.5 [45.4-181.4]	94.3 [41.5-149]	0.618
Time to Death, hours	534	189 [106-377]	190 [103-371]	182 [114-404]	0.618
Time to Intubation or Death, hours	695	123 [63-230]	122 [62-227]	128 [71-270]	0.211

Data are presented as n (%), median [IQR], or mean ± SD.

S5 Table. Comparison of primary analysis patients with patients excluded for having all inflammatory markers missing in the external validation cohort (Cornell wild type wave).

	No. available	All	Included: 1-5 inflammatory biomarkers	Excluded: inflammatory markers missing	p-value
**No. patients**	1453	1453	1214	239	
**Demographics**					
Age, yr	1453	67 [54-78]	67 [56-79]	60 [43-72]	<0.001
Male sex	1453	818 (56)	697 (57)	121 (51)	0.063
Body mass index	1318	28 ± 7.2	28 ± 7.2	28 ± 7.2	0.706
Race	1453				
Black		198 (14)	157 (13)	41 (17)	0.102
White		420 (29)	345 (28)	75 (31)	0.398
Other/Unknown		834 (57)	712 (59)	122 (51)	0.036
Hispanic	1453	360 (25)	299 (25)	61 (26)	0.833
**Comorbidities**					
Any Below Comorbidity	1453	1104 (76)	955 (79)	149 (62)	<0.001
Cancer	1453	409 (28)	343 (28)	66 (28)	0.903
Chronic kidney disease	1453	290 (20)	253 (21)	37 (15)	0.071
Chronic heart failure	1453	233 (16)	206 (17)	27 (11)	0.037
Diabetes (without complications)	1453	571 (39)	487 (40)	84 (35)	0.172
Diabetes (with complications)	1453	369 (25)	328 (27)	41 (17)	0.002
Essential hypertension	1453	882 (61)	767 (63)	115 (48)	<0.001
Smoker (current or former)	1453	442 (30)	386 (32)	56 (23)	0.013
Charlson comorbidity index	1416	2 [1-5]	2 [1-5]	1 [0-4]	0.002
**Laboratory**					
Albumin, g/dL	1378	3.3 ± 0.6	3.3 ±0.6	3.7 ± 0.6	<0.001
Alanine aminotransferase, U/L	1378	32 [20-52]	33 [20-54]	26 [18-40]	<0.001
Aspartate aminotransferase, U/L	731	39 [27-60]	41 [27-62]	31 [23-45]	0.001
Bilirubin, mg/dL	1378	0.6 [0.4-0.9]	0.6 [0.4-0.9]	0.5 [0.4-0.8]	0.005
Hemoglobin, g/dL	1451	13.1 ± 2.1	13.1 ± 2.1	13.0 ± 2.1	0.451
WBC, x10^9/L	1436	6.8 [5.1-9.4]	6.8 [5.0-9.5]	6.9 [5.1-9.0]	0.791
Lymphocyte count x10^9/L	1387	0.8 [0.6-1.1]	0.8 [0.6-1.1]	0.9 [0.7-1.4]	<0.001
% Neutrophils	878	76 [67-84]	77 [68-84]	70 [58-78]	<0.001
Platelets, x10^9/L	1448	204 [156-268]	206 [155-272]	201 [164-253]	0.722
D-dimer, ug/L FEU	847	537 [310-1073]	546 [312-1073]	356 [206-831]	0.079
International Normalized Ratio	1153	1.1 [1.0-1.2]	1.2 [1.1-1.3]	1.1 [1.0-1.2]	0.002
aPTT, second(s)	1107	32 [29-34]	32 [29-34]	32 [29-34]	0.961
BUN, mg/dL	1423	17 [12-28]	17 [12-29]	17 [12-24]	0.148
Creatinine, mg/dL	1070	0.89 [0.70-1.28]	0.90 (0.70-1.28)	0.86 [0.68-1.20]	0.430
Bicarbonate, mmol/L	1423	25 [22-27]	25 (22-27)	25 [22-27]	0.264
Sodium, mmol/L	1423	137 [134-140]	137 [134-140]	138 [136-140]	<0.001
Troponin-I, ng/L	1157	0.03	0.03	0.03	0.003

		[0.03-0.05]	[0.03-0.05]	[0.00-0.03]	
**Vitals**					
Mean Arterial Pressure, mmHg	1443	95 [86-104]	95 [86-104]	93 [86-103]	0.961
Temperature, ˚C	1443	37.3 ± 0.8	37.3 ± 0.8	37.2 ± 0.8	0.003
Heart Rate, /min	1443	96 ± 19	97 ± 19	94 ± 19	0.048
P_aO_2/F_iO_2 ratio	1441	211 [81-303]	193 [76-279]	304 [213-360]	<0.001
Respiratory Rate, per min	1443	18 [18-22]	20 [18-22]	18 [18-20]	<0.001
**Outcomes**					
Intubation	1453	269 (19)	247 (20)	22 (9.2)	0.001
Death at 90 days	1453	160 (11)	146 (12)	14 (5.8)	0.008
Intubation or Death	1453	362 (25)	331 (27)	31 (13)	<0.001
DNR history	NA	NA	NA	NA	NA
Time to Intubation, hours	278	77.1 [46.3-124]	75.3 [45.5-120]	105 [56.3-162]	0.219
Time to Death, hours	218	251 [136-803]	249 [139-790]	266 [119-2501]	0.994
Time to Intubation or Death, hours	396	94.1 [51.5-174]	89.5 [50.8-169]	119 [73-217]	0.140

Data are presented as n (%), median [IQR], or mean ± SD.

S6 Table. Comparison of primary analysis patients with patients excluded for having all inflammatory markers missing in the longitudinal validation cohort (Columbia delta wave).

	No. available	All	Included: 1-5 inflammatory biomarkers	Excluded: inflammatory markers missing	p-value
**No. patients**	2094	2094	1536	558	
**Demographics**					
Age, yr	2094	67 (54-78)	67 (54-78)	67 (53-78)	0.449
Male Sex	2094	1069 (51)	785 (51)	284 (51)	0.971
Body Mass Index	1816	29 ± 6.7	29 ± 6.8	28 ± 6.4	0.012
Race	2094				
Black		351 (17)	243 (16)	108 (19)	0.065
White		515 (25)	391 (26)	124 (22)	0.144
Other/Unknown		1228 (59)	902 (59)	326 (58)	0.941
Hispanic	2094	1100 (53)	819 (53)	281 (50)	0.250
**Comorbidities**					
Any Below Comorbidity	2094	1315 (63)	971 (63)	344 (62)	0.545
Cancer	NA	NA	NA	NA	
Chronic kidney disease	2094	379 (18)	283 (18)	96 (17)	0.564
Chronic heart failure	2094	335 (16)	256 (17)	79 (14)	0.188
Diabetes (without complications)	2094	770 (37)	579 (38)	191 (34)	0.161
Diabetes (with complications)	2094	481 (23)	347 (23)	134 (24)	0.532
Essential hypertension	2094	1106 (53)	811 (53)	295 (53)	0.999
Smoker (current or former)	2094	277 (13)	208 (14)	69 (12)	0.530
Charlson comorbidity index	2094	1 [0-4]	1 [0-4]	1 [0-3]	0.568
**Laboratory**					
Albumin, g/dL	1932	3.8 ± 0.5	3.7 ± 0.5	3.8 ± 0.6	0.001
Alanine aminotransferase, U/L	1922	26 [17-44]	27 [17-45]	24 [16-41]	0.004
Aspartate aminotransferase, U/L	1662	39 [27-62]	40 [28-63]	34 [24-58]	<0.001
Bilirubin, mg/dL	1931	0.4 [0.3-0.6]	0.4 [0.3-0.6]	0.4 [0.3-0.6]	0.876
Hemoglobin, g/dL	2070	12.8 ± 2.2	12.7 ± 2.2	12.9 ± 2.3	0.073
WBC, x10^9/L	2054	6.5 [4.9-8.7]	6.5 [4.9-8.7]	6.5 [5.0-8.7]	0.776
Percent Lymphocytes	1972	13 [7.0-21]	14 [8.0-21]	13 [4.0-21]	0.002
Percent Neutrophils	1639	71 [58-80]	71 [55-80]	72 [62-79]	0.189
Neutrophil-Lymphocyte Ratio	1627	4.0 [2.1-7.0]	3.9 [1.9-6.8]	4.3 [2.6-6.8]	0.063
Platelets, x10^9/L	2054	201 [155-268]	200 [156-267]	204 [153-273]	0.636
D-dimer, ug/mL FEU	1195	1.2 [0.69-2.2]	1.2 [0.70-2.2]	1.1 [1.0-1.2]	0.312
International Normalized Ratio	1436	1.1 [1.0-1.2]	1.1 [1.0-1.2]	1.1 [1.0-1.2]	0.098
aPTT, second[s]	1433	33 [30-36]	33 [30-36]	33 [29-36]	0.153
BUN, mg/dL	2058	17 [12-28]	17 [11-28]	18 [12-28]	0.108
Creatinine, mg/dL	2062	1.1 [0.86-1.50]	1.1 [0.86-1.50]	1.11 [0.84-1.53]	0.691
Bicarbonate, mmol/L	2058	23 [21-26	24 [21-26]	23 [21-26]	0.146

Sodium, mmol/L	2061	137 [134-140]	137 [34-140]	137 [134-140]	0.948
Troponin-T, ng/L	1616	16 [9-33]	16 [8-32]	18 [10-38]	0.013
**Vitals**					
Mean Arterial Pressure, mmHg	2090	93 [85-103]	93 [85-102]	95 [85-105]	0.012
Temperature, ˚F	2090	99.2 ± 1.4	99.3 ± 1.5	98.9 ± 1.3	<0.001
Hear Rate, /min	2092	96 ± 19	97 ±19	95 ±19	0.049
P_aO_2/F_iO_2 ratio	2065	21 [84-291]	200 [76-269]	260 [163-338]	<0.001
Respiratory Rate, /min	2094	18 [18-20]	19 [18-20]	18 [18-20]	<0.001
SOFA score	1661	2 [1-3]	2 [1-3]	2 [1-3]	0.008
**Outcomes**					
Intubation	2094	77 (3.6)	64 (4.1)	13 (2.3)	0.065
Death at 90 days	2094	280 (13)	224 (15)	56 (10)	0.009
Intubation or Death	2094	305 (15)	247 (16)	58 (10)	0.001
DNR history	2094	82 (3.9)	63 (4.1)	19 (3.4)	0.549
Time to Intubation, hours	77	168 [98.7-312]	164 [97.8-313]	225 [115-300]	0.678
Time to Death, hours	278	412 [237-783]	415 [230-833]	405 [260-560]	0.297
Time to Intubation or Death, hours	309	312 [162-529]	308 [160-531]	324 [181-517]	0.977

Data are presented as n (%), median [IQR], or mean ± SD.

S7 Table. Average latent class membership probabilities for the latent class models described in Table 1.

Class	Average latent class membership probabilities				
**Columbia Wild Type Wave (March 2020-June 2020)**					
	1	2	3	4	5
2	0.96	0.94			
3	0.90	0.90	0.93		
4	0.90	0.89	0.87	0.92	
5	0.86	0.89	0.89	0.92	0.94
**Cornell Wild Type Wave (March 2020-June 2020)**					
Class	Average latent class membership probabilities				
	1	2	3	4	5
2	0.93	0.92			
3	0.93	0.90	0.90		
4	0.93	0.96	0.90	0.90	
5	0.97	0.88	0.91	0.91	0.95
**Columbia Delta Wave (October 2020-June 2021)**					
Class	Average latent class membership probabilities				
	1	2	3	4	5
2	0.94	0.91			
3	0.90	0.92	0.90		
4	0.89	0.91	0.91	0.90	
5	0.87	0.87	0.87	0.93	0.88

S8 Table. Latent class model fit statistics for sensitivity analyses including patients without any inflammatory biomarkers (CRP, Ferritin, ESR, LDH, IL-6) measured in the first 24 hours after hospital admission.

Columbia Wild Type Wave (March 2020 - June 2020), n = 2,410								
Classes	BIC	Entropy	VLMR p-value	Individuals per Latent Class				
				1	2	3	4	5
2	154984	0.82	<0.001	1567	843			
3	153495	0.78	<0.001	875	780	755		
4	152315	0.8	0.01	630	639	754	387	
5	151272	0.82	0.657	609	630	737	59	375
Classes	Average Latent Class Membership Probabilities							
	1	2	3	4	5			
2	0.96	0.93						
3	0.90	0.89	0.93					
4	0.87	0.89	0.89	0.92				
5	0.85	0.88	0.88	0.92	0.91			
**Cornell Wild Type Wave (March 2020 - June 2020), n = 1,453**								
Classes	BIC	Entropy	VLMR p-value	Individuals per Latent Class				
				1	2	3	4	5
2	91884	0.73	<0.001	899	554			
3	91047	0.8	<0.001	822	393	238		
4	90407	0.84	0.008	28	223	396	806	
5	89952	0.84	0.328	254	395	730	25	49
Classes	Average Latent Class Membership Probabilities							
	1	2	3	4	5			
2	0.93	0.91						
3	0.93	0.89	0.91					
4	0.94	0.90	0.89	0.93				
5	0.87	0.89	0.91	0.97	0.97			
**Columbia Delta Wave (October 2020 - June 2021), n = 2,094**								
Classes	BIC	Entropy	VLMR p-value	Individuals per Latent Class				
				1	2	3	4	5
2	129482	0.74	<0.001	1246	848			
3	128221	0.75	0.035	782	773	539		
4	127282	0.79	0.092	752	736	84	522	
5	126664	0.78	0.571	683	444	516	376	75
Classes	Average Latent Class Membership Probabilities							
	1	2	3	4	5			
2	0.94	0.90						
3	0.88	0.90	0.89					
4	0.87	0.89	0.91	0.88				
5	0.86	0.85	0.86	0.86	0.91			

S9 Table. Latent class model fit statistics for sensitivity analyses excluding variables with >25% missingness.

Columbia Wild Type Wave (March 2020 - June 2020), n = 2,077, BMI, D-Dimer, and IL-6 excluded								
Classes	BIC	Entropy	VLMR p-value	Individuals per Latent Class				
				1	2	3	4	5
2	128093	0.81	0.018	1318	759			
3	126708	0.79	0.011	726	699	652		
4	125787	0.82	0.002	685	620	711	61	
5	125022	0.82	0.379	298	443	698	587	51
Classes	Average Latent Class Membership Probabilities							
	1	2	3	4	5			
2	0.96	0.93						
3	0.91	0.88	0.94					
4	0.89	0.91	0.90	0.89				
5	0.91	0.86	0.89	0.88	0.94			
**Cornell Wild Type Wave (March 2020 - June 2020), n = 1,214, D-Dimer and ESR excluded**								
Classes	BIC	Entropy	VLMR p-value	Individuals per Latent Class				
				1	2	3	4	5
2	75574	0.74	<0.001	703	511			
3	74867	0.80	<0.001	638	370	206		
4	74322	0.84	0.009	632	23	370	189	
5	73964	0.84	0.350	22	554	217	370	51
Classes	Average Latent Class Membership Probabilities							
	1	2	3	4	5			
2	0.93	0.91						
3	0.94	0.88	0.90					
4	0.92	0.98	0.90	0.90				
5	0.99	0.90	0.87	0.90	0.94			
**Columbia Delta Wave (October 2020 - June 2021), n = 1,536, D-Dimer, IL-6, ESR, LDH excluded**								
Classes	BIC	Entropy	VLMR p-value	Individuals per Latent Class				
				1	2	3	4	5
2	91321	0.75	<0.001	908	628			
3	90448	0.77	<0.001	639	541	356		
4	89808	81	0.2117	588	519	66	363	
5	89388	0.84	<0.001	523	450	159	61	343
Classes	Average Latent Class Membership Probabilities							
	1	2	3	4	5			
2	0.94	0.91						
3	0.91	0.89	0.89					
4	0.91	0.88	0.91	0.89				
5	0.9.0	0.88	0.95	0.94	0.88			

S10 Table. Latent class model fit statistics for sensitivity analyses excluding variables associated with in-hospital death.

**Columbia Wild Type Wave (March 2020 - June 2020), n = 2,077, BMI, D-Dimer, and IL-6 excluded**								
Classes	BIC	Entropy	VLMR p-value	Individuals per Latent Class				
				1	2	3	4	5
**2**	128093	0.81	0.018	1318	759			
**3**	126708	0.79	0.011	726	699	652		
**4**	125787	0.82	0.002	685	620	711	61	
**5**	125022	0.82	0.379	298	443	698	587	51
Classes	Average Latent Class Membership Probabilities							
	1	2	3	4	5			
**2**	0.99	0.93						
**3**	0.91	0.88	0.94					
**4**	0.89	0.91	0.90	0.89				
**5**	0.91	0.86	0.89	0.88	0.94			
**Cornell Wild Type Wave (March 2020 - June 2020), n = 1,214, troponin excluded**								
Classes	BIC	Entropy	VLMR p-value	Individuals per Latent Class				
				1	2	3	4	5
**2**	77824	0.74	<0.001	691	523			
**3**	77253	0.83	0.0060	680	510	24		
**4**	76748	0.83	0.0510	228	607	356	23	
**5**	76354	0.81	0.2700	86	393	343	370	22
Classes	Average Latent Class Membership Probabilities							
	1	2	3	4	5			
**2**	0.94	0.91						
**3**	0.93	0.91	0.98					
**4**	0.87	0.93	0.89	0.97				
**5**	0.92	0.87	0.87	0.89	1.00			
**Columbia Delta Wave (October 2020 - June 2021), n = 1,536, BMI, troponin, INR, APTT excluded**								
Classes	BIC	Entropy	VLMR p-value	Individuals per Latent Class				
				1	2	3	4	5
**2**	87119	0.76	<0.001	966	570			
**3**	86394	0.77	<0.001	692	481	363		
**4**	86021	0.75	0.005	356	347	449	384	
**5**	85752	0.79	0.181	325	212	522	394	83
Classes	Average Latent Class Membership Probabilities							
	1	2	3	4	5			
**2**	0.94	0.92						
**3**	0.90	0.91	0.87					
**4**	0.86	0.89	0.84	0.85				
**5**	0.85	0.85	0.87	0.87	0.92			

S11 Table. Latent class model fit statistics for sensitivity analyses with estimated PaO2:FiO2 at ED triage via back fill imputation of FiO2 for Columbia wildtype and delta wave cohorts.

**Columbia Wild Type Wave (March 2020 - June 2020), n = 2,077, imputed PaO2:FiO2 at ED triage**								
Classes	BIC	Entropy	VLMR p-value	Individuals per Latent Class				
				1	2	3	4	5
**2**	138726	0.82	<0.001	1316	763			
**3**	137341	0.8	<0.001	676	720	683		
**4**	136326	0.81	0.0286	520	512	695	352	
**5**	135479	0.83	0.0239	346	505	674	503	51
Classes	Average Latent Class Membership Probabilities							
	1	2	3	4	5			
**2**	0.96	0.93						
**3**	0.93	0.9	0.91					
**4**	0.87	0.9	0.89	0.92				
**5**	0.922	0.864	0.892	0.892	0.94			
**Columbia Delta Wave (October 2020 - June 2021), n = 1,536, imputed PaO2:FiO2 at ED triage**								
Classes	BIC	Entropy	VLMR p-value	Individuals per Latent Class				
				1	2	3	4	5
**2**	101204	0.764	**0.00010**	943	593			
**3**	100071	0.784	**0.00040**	537	562	437		
**4**	99451	0.82	0.33340	65	530	513	428	
**5**	99001	0.805	0.36040	414	321	422	326	53
Classes	Average Latent Class Membership Probabilities							
	1	2	3	4	5			
**2**	0.941	0.914						
**3**	0.896	0.91	0.904					
**4**	0.901	0.905	0.885	0.909				
**5**	0.863	0.876	0.868	0.884	0.929			

S12 Table. Characteristics of hospitalized adults with non-critical COVID-19 by the 3-class subphenotype model (Columbia wild type wave).

	No. available	All	Low-Inflammatory	Intermediate-Inflammatory	High-Inflammatory with fibrinolysis	p-value
No. patients	2077	2077	692	708	677	
**Comorbidities**						
Any Below Comorbidity	2077	1439 (69)	497 (72)	400 (57)	542 (80)	<0.001
Cancer	2077	354 (17)	119 (17)	78 (11)	157 (23)	<0.001
Chronic kidney disease	2077	435 (21)	145 (21)	40 (5.6)	250 (37)	<0.001
Chronic heart failure	2077	355 (17)	129 (19)	51 (7.2)	175 (26)	<0.001
Diabetes(without complications)	2077	824 (40)	273 (40)	223 (32)	328 (48)	<0.001
Diabetes (with complications)	2077	485 (23)	169 (24)	87 (12)	229 (34)	<0.001
Essential hypertension	2077	1141 (55)	430 (62)	279 (39)	432 (64)	<0.001
Smoker (current or former)	2077	259 (13)	97 (14)	48 (6.8)	114 (17)	<0.001
**Laboratory**						
Albumin, g/dL	2000	3.7 ± 0.5	3.9 ± 0.5	3.8 ± 0.4	3.4 ± 0.5	<0.001
ALT, U/L	1982	29 [18-50]	22 [15-32]	42 [27-66]	27 [17-50]	<0.001
Bilirubin, mg/dL	1993	0.5 [0.3-0.7]	0.4 [0.3-0.6]	0.5 [0.4-0.7]	0.5 [0.4-0.8]	<0.001
Hemoglobin, g/dL	2060	12.9 ± 2.3	12.6 ± 2.2	13.8 ± 1.7	12.3 ± 2.7	<0.001
WBC, x10^9/L	2043	7.3 [5.5-10]	5.66 [4.6-7.2]	7.5 [6.0-9.8]	9.4 [7.1-13.1]	<0.001
Percent Lymphocytes	2035	13 [8.6-20]	19 [14-25]	13 [9.1-18]	9.0 [6.0-13]	<0.001
Percent Neutrophils	1973	75 [64-83]	69 [60-76]	77 [68-83]	81 [70-86]	<0.001
Neutrophil-Lymphocyte Ratio	1958	5.0 [2.7-8.7]	3.4 [2.1-5.3]	5.3 [3.2-8.4]	7.8 [4.3-13]	<0.001
Platelets, x10^9/L	2049	203 [155-265]	183 [144-242]	214 [167-272]	215 [159-287]	<0.001
D-dimer, ug/mL FEU	1268	1.37 [0.77-2.81]	0.98 [0.56-1.79]	1.05 [0.70-1.75]	3.14 [1.75-10.9]	<0.001
International Normalized Ratio	1678	1.1 [1.0-1.2]	1.1 [1.0-1.2]	1.1 [1.0-1.1]	1.2 [1.1-1.3]	<0.001
APTT, seconds	1665	33 [30-37]	32 [30-35]	33 [30-36]	34 [30-38]	<0.001
BUN, mg/dL	2066	19 [12-35]	16 [11-24]	13 [10-18]	43 [28-69]	<0.001
Creatinine, mg/dL	2066	1.08 [0.82-1.67]	0.97 [0.77-1.36]	0.93 [0.75-1.1]	1.8 [1.2-3.4]	<0.001
Bicarbonate, mmol/L	2065	232 [20-25]	24 [21-26]	23 [21-25]	20 [17-23]	<0.001
Sodium, mml/L	2066	137 [134-140]	138 [135-140]	136 [133-139]	138 [135-144]	<0.001
Troponin-T, ng/L	1825	17 [8-40]	15 [8-26]	9 [6-13]	50 [27-110]	<0.001
Interleukin-6, pg/mL	907	33.4 [11.0-80.1]	10.5 [5.0-26.9]	44 [15-78]	83 [39-158]	<0.001
C-Reactive Protein, mg/L	1900	116 [60-196]	49 [14-84]	148 [94-204]	178 [114-274]	<0.001
Lactate Dehydrogenase, U/L	1866	410 [306-561]	298 [230-367]	463 [374-584]	506 383-691]	<0.001
Ferritin, ng/mL	1826	696.9 [342-1254]	315 [156-599]	891 [529-1475]	928 [490-1755]	<0.001

Erythrocyte Sedimentation Rate, mm/hr	1594	72 [50-97]	52 [33-74]	76 [58-97]	87 [64-111]	<0.001
**Vitals**						
Mean Arterial Pressure, mmHg	2076	92 [82-101]	93 [83-102]	94 [86-101]	89 [78-100]	<0.001
Temperature, ºF	2076	99.3 ± 1.6	99.2 ± 1.4	99. ± 1.7	98.9 ± 1.5	<0.001
Heart Rate, per min	2075	98 ± 19	92 ± 18	106 ± 17	96 ± 21	<0.001
P_aO_2/F_iO_2 ratio	2055	150 [64-226]	229 [168-304]	94.6 [62.5-203]	70.7 [51.0-159]	<0.001
Respiratory Rate, per min	2073	20 [18-22]	18 [18-20]	20 [18-24]	20 [18-24]	<0.001
**Outcomes**						
DNR history	2077	103 (4.9)	48 (6.9)	2 (0.3)	53 (7.8)	<0.001
Intubation or Death	2077	623 (30)	85 (12)	173 (24)	365 (54)	<0.001
Time to Intubation, hours	299	87.5 [45.4-181.4]	132 [68.0-419]	107 [48.7-197]	66.1 [41.2-142]	0.007
Time to Death, hours	476	190 [103-371]	253 [151-419]	373 [182-703]	156 [86.7-274]	<0.001
Time to Intubation or Death, hours	628	122 [62.0-227]	185 [116-334]	115 [56.2-228]	115 [57.3-202]	<0.001

Data are presented as n (%), median [IQR], or mean ± SD.

S13 Table. Characteristics of hospitalized adults with non-critical COVID-19 by the 3-class subphenotype model (Cornell wild type wave).

	No. available	All	Low-Inflammatory	Intermediate-Inflammatory	High-Inflammatory with fibrinolysis	p-value
Number of Patients	1214	1214	630	376	208	
**Comorbidities**						
Any Below Comorbidity	1214	955 (79)	480 (76)	282 (75)	193 (93)	0.00
Cancer	1214	343 (28)	180 (29)	90 (24)	73 (35)	0.02
CKD	1214	253 (21)	109 (17)	48 (13)	96 (46)	0.00
CHF	1214	206 (17)	94 (15)	33 (8.8)	79 (38)	0.00
Diabetes (without complications)	1214	487 (40)	264 (42)	151 (40)	72 (35)	0.18
Diabetes (with complications)	1214	328 (27)	154 (24)	96 (26)	78 (38)	0.00
Essential hypertension	1214	767 (63)	390 (62)	234 (62)	143 (69)	0.19
Smoker (current or former)	1214	386 (32)	190 (30)	126 (34)	70 (34)	0.44
**Laboratory**						
Albumin, g/dL	1192	3.3 ± 0.59	3.4 ± 0.6	3.1 ± 0.5	3.0 ± 0.6	<0.001
ALT, U/L	1192	33 [20-54]	26 [17-41]	54 [36-92]	28 [18-45]	<0.001
Bilirubin, mg/dL	1192	0.6 [0.4-0.9]	0.5 [0.4-0.7]	0.7 [0.6-1.0]	0.7 [0.4-1.2]	<0.001
Hemoglobin, g/dL	1214	13.1 ± 2.1	13.0 ± 1.9	13.8 ± 1.7	12.3 ± 2.6	<0.001
WBC, $x10^9/L$	1200	6.8 [5.0-9.5]	5.7 [4.34-7.4]	8.7 [6.6-10.8]	8.0 [5.9-11]	<0.001
Lymphocyte count $x10^9/L$	1185	0.8 [0.6-1.1]	0.9 [0.6-1.2]	0.8 [0.6-1.1]	0.7 [0.5-1.1]	<0.001
Percent Neutrophils	782	77 (68-84]	71 [62-79]	81 [76-87]	83 [77-89]	<0.001
Platelets, $x10^9/L$	1211	206 [155-272]	191 [148-248]	236 [187-305]	189 [133-258]	<0.001
D-dimer, ng/mL FEU	821	546 [312-1073]	452 [272-796]	501 [320-942]	2012 [690-3275]	<0.001
International Normalized Ratio	1031	1.2 [1.1-1.3]	1.1 [1.0-1.2]	1.0 [1.1-1.3]	1.2 [1.1-14]	<0.001
APTT, seconds	996	32 [29-34]	31 [29-34]	32 [29-34]	33 [30-37]	0.026
BUN, mg/dL	1212	17 [12-29]	15 [11-22]	16 [12-23]	48 [34-74]	<0.001
Creatinine, mg/dL	948	0.90 [0.7-1.3]	0.81 [0.67-1.1]	0.81 [0.70-1.0]	1.9 [1.2-3.8]	<0.001
Bicarbonate, mmol/L	1212	25 [22-27]	25 [23-28]	26 [23-27]	21 [18-25]	<0.001
Sodium, mml/L	1212	137 [134-140]	137 [134-139]	136 [133-139]	138 [135-143]	<0.001
Troponin-I, ng/L	1047	0.03 [0.03-0.05]	0.03 [0.03-0.03]	0.03 [0.03-0.03]	0.11 [0.06-0.31]	<0.001
Interleukin-6, pg/mL	115	17 [7.0-46]	8.5 [5.0-19]	17 [5-51]	39 [13.8-110]	<0.001
C-Reactive Protein, mg/L	915	9.3 [4.8-16]	5.9 [2.8-9.3]	18 [13-26]	12 [7.8-18]	<0.001
Lactate Dehydrogenase, U/L	1108	399 [298-523]	317 [252-390]	514 [426-631]	475 [370-650]	<0.001
Ferritin, ng/mL	1009	721 [336-1449]	398 [183-729]	1280 [704-1912]	1116 [652-2000]	<0.001
Erythrocyte Sedimentation Rate, mm/hr	840	70 [45-95]	57 [38-80]	84 [62-105]	74 [44-105]	<0.001
**Vitals**						
Mean Arterial Pressure, mmHg	1214	95 [86-104]	95 [86-105]	98 [89-106]	88 [78-96]	<0.001

Temperature, °F	1214	37.3 ± 0.8	37.3 ± 0.8	37.5 ± 0.9	37.0 ± 0.8	<0.001
Heart Rate, per min	1214	97 ± 19	94 ± 19	102 ± 18	96 ± 21	<0.001
P_aO_2/F_iO_2 ratio	1214	193 [76-279]	257 [172-319]	94.9 [60.9-198]	101 [64-215]	<0.001
Respiratory Rate, per min	1214	20 [18-22]	18 [18-20]	20 [18-24]	20 [18-25]	<0.001
**Outcomes**						
Intubation or Death	1214	329 (27)	90 (15)	132 (35)	107 (51)	<0.001
Time to Intubation, hours	256	75.3 [45.5-120]	85.1 [50.7-135]	75.2 [43.3-111]	71.6 [41.6-134.6]	0.28
Time to Death, hours	199	249 [139-790]	402 [209-4260]	448 [194-820]	424 [83-424]	<0.001
Time to Intubation or Death, hours	360	89.5 [50.8-169]	125 [59.4-266]	75.3 [43.6-113]	97.7 [56.7-186]	<0.001

Data are presented as n (%), median [IQR], or mean ± SD.

S14 Table. Characteristics of hospitalized adults with non-critical COVID-19 by the 3-class subphenotype model (Columbia delta wave).

	No. available	All	Low-Inflammatory	Intermediate-Inflammatory	High-Inflammatory with fibrinolysis	p-value
Number of Patients	1536	1536	541	557	438	
**Comorbidities**						
Any Below Comorbidity	1536	971 (63)	436 (81)	238 (43)	297 (68)	<0.001
Cancer	NA	NA	NA	NA	NA	
CKD	1536	283 (18)	177 (33)	23 (4.1)	83 (19)	<0.001
CHF	1536	256 (17)	152 (28)	30 (5.4)	74 (17)	<0.001
Diabetes (without complications)	1536	579 (38)	265 (49)	139 (25)	175 (40)	<0.001
Diabetes (with complications)	1536	347 (23)	169 (31)	61 (11)	117 (27)	<0.001
Essential hypertension	1536	811 (53)	389 (72)	182 (33)	240 (55)	<0.001
Smoker (current or former)	1536	208 (14)	107 (20)	57 (10)	44 (10)	<0.001
**Laboratory**						
Albumin, g/dL	1482	3.7 ± 0.5	3.7 ± 0.5	4.0 ± 0.4	3.5 ± 0.4	<0.001
ALT, U/L	1475	27 [17-45]	19 [13-27]	35 [23-62]	31 [20-58]	<0.001
Bilirubin, mg/dL	1481	0.4 [0.3-0.6]	0.4 [0.3-0.6]	0.4 [0.3-0.6]	0.5 [0.3-0.7]	<0.001
Hemoglobin, g/dL	1533	12.7 ± 2.2	11.7 ± 2.2	13.7 ± 1.9	12.8 ± 2.1	<0.001
WBC, $x10^9/L$	1520	6.5 [4.9-8.7]	5.7 [4.4-7.5]	5.9 [4.5-7.6]	8.8 [6.7-12]	<0.001
Percent Lymphocytes	1467	13.7 [8.0-21]	17 [11-24]	17 (11-23]	8.5 [4.4-13]	<0.001
Percent Neutrophils	1268	71 [55-80]	69 [57-77]	69 [52-77]	80 [12-85]	<0.001
Neutrophil-Lymphocyte Ratio	1258	3.9 [1.9-7.1]	3.8 [2.1-5.4]	3.2 [1.6-5.4]	6.5 [2.6-11]	<0.001
Platelets, $x10^9/L$	1521	200 [156-267]	181 [140-243]	195 [159-247]	242 [176-318]	<0.001
D-dimer, ug/mL FEU	1098	1.2 [0.70-2.2]	1.2 [0.77-2.3]	0.78 [0.5-1.2]	2.0 [1.2-3.4]	<0.001
International Normalized Ratio	1180	1.1 [1.0-1.2]	1.1 [1.0-1.2]	1.0 [1.0-1.1]	1.1 [1.1-1.2]	<0.001
APTT, seconds	1176	33 [30-36]	33 [30-37]	32 [29-35]	34 [30-37]	<0.001
BUN, mg/dL	1531	17 [11-28]	24 [16-36]	11 [9-15]	21 [15-34]	<0.001
Creatinine, mg/dL	1533	1.1 [0.86-1.5]	1.33 [1.0-2.0]	0.93 [0.77-1.12]	1.19 [0.92-1.7]	<0.001
Bicarbonate, mmol/L	1532	24 [21-26]	24 [21-26]	24 [22-26]	22 [20-25]	<0.001
Sodium, mml/L	1533	137 [134-140]	137 [134-140]	137 [135-139]	136 [133-140]	0.004
Troponin-T, ng/L	1273	16 [8-32]	27 [17-51]	8 [6-11]	21 [12-36]	<0.001
Interleukin-6, pg/mL	397	34.8 [15.7-77]	23 [12-56]	26 [12-53]	78 [37-128]	<0.001
C-Reactive Protein, mg/L	1372	845 [37-155]	50 [16-93]	66 [30-107]	190 [143-243]	<0.001
Lactate Dehydrogenase, U/L	1148	374 [281-522]	294 [233-368]	369 [290-491]	508 [401-681]	<0.001
Ferritin, ng/mL	1207	545 [258-1089]	351 [161-692]	529 [290-1067]	902 [491-1546]	<0.001
Erythrocyte Sedimentation Rate, mm/hr	961	58 [38-85]	49 [29-74]	51 [33-69]	84 [64-107]	<0.001
**Vitals**						

Mean Arterial Pressure, mmHg	1533	93 [85-102]	92 [83-103]	94 [86-102]	92 [85-101]	0.157
Temperature, °F	1533	99.3 ± 1.5	98.9 ± 1.2	99.8 ± 1.5	99 ± 1.5	<0.001
Heart Rate, per min	1535	97 ± 19	86 ± 16	103 ± 16	102 ± 20	<0.001
P_aO_2/F_iO_2 ratio	1536	200 [76-269]	236 [171-304]	217 [134-280]	76.0 [47-189]	<0.001
Respiratory Rate, per min	1536	19 [18-20]	18 [18-20]	19 [18-20]	20 [18-24]	<0.001
**Outcomes**						
DNR history	1536	63 (4.1)	36 (6.7)	4 (0.7)	23 (5.2)	<0.001
Intubation or Death	1536	247 (16)	70 (13)	37 (6.6)	140 (32)	<0.001
Time to Intubation, hours	64	164 [97.8-313]	114 [71.6-349]	261 [164-333]	143 [87.7-232]	0.024
Time to Death, hours	222	415 [230-833]	414 [240-845]	623 [364-1099]	355 [204-787]	0.029
Time to Intubation or Death, hours	250	308 [150-531]	338 [158-569]	316 [245-441]	274 [149-524]	0.335

Data are presented as n (%), median [IQR], or mean ± SD.

S15 Table. Restricted mean survival time (RMST) at 90 days

Columbia Wild Type Wave (March 2020 - June 2020)	RMST (95% CI), %	RMST Difference (95% CI), %	p-value
Low-Inflammatory	79 (77-81)	Reference	
Intermediate-Inflammatory	69 (66-72)	minus 10 (-13 to -7)	<0.001
High-Inflammatory with Fibrinolysis	45 (42-48)	minus 34 (-38 to -31)	<0.001
**Cornell Wild Type Wave (March 2020 - June 2020)**			
Low-Inflammatory	78 (75-80)	Reference	
Intermediate-Inflammatory	60 (56-64)	minus 18 (-23 to -13)	<0.001
High-Inflammatory with Fibrinolysis	46 (40-52)	minus 32 (-38 to -25)	<0.001
**Columbia Delta Wave (October 2020 - June 2021)**			
Low-Inflammatory	81 (79-83)	Reference	
Intermediate-Inflammatory	85 (84-86)	4 (1.8 to 7.0)	0.001
High-Inflammatory with Fibrinolysis	66 (63-70)	minus 14 (-18 to -10)	<0.001

S16 Table. Characteristics of hospitalized adults with non-critical COVID-19 from the Columbia wild type wave cohort by the 2-class subphenotype model

	No. available	Hypoinflammatory	Hyperinflammatory	p-value
Number of Patients	2077	1320	757	
**Demographics**				
Age, years	2077	62 [51-73]	75 [66-84]	<0.001
Male sex	2077	726 (55)	464 (61)	0.006
Body Mass Index	1709	30 ± 6.9	27 ± 6.1	<0.001
Race	2077			0.151
Black		245 (19)	167 (22)	0.062
White		304 (23)	163 (22)	0.464
Other/Unknown		771 (58)	427 (56)	0.399
Hispanic Ethnicity	2077	678 (51)	366 (48)	0.202
**Comorbidities**				
Any Below Comorbidity	2077	841 (64)	598 (79)	<0.001
Cancer	2077	190 (14)	164 (22)	<0.001
Chronic kidney disease	2077	180 (14)	255 (34)	<0.001
Chronic heart failure	2077	174 (13)	181 (24)	<0.001
Diabetes (without complications)	2077	470 (36)	354 (47)	<0.001
Diabetes (with complications)	2077	242 (18)	243 (32)	<0.001
Essential hypertension	2077	667 (50)	474 (63)	<0.001
Smoker (current or former)	2077	142 (11)	117 (16)	0.002
Charlson comorbidity Index	2077	0 [1-3]	2 [1-5]	<0.001
**Laboratory**				
Albumin, g/dL	2000	3.90 ± 0.4	3.4 ± 0.5	<0.001
ALT, U/L	1982	29 [19-47]	29 [18-53]	0.665
Bilirubin, mg/dL	1993	0.5 [0.3-0.6]	0.5 [0.4-0.8]	<0.001
Hemoglobin, g/dL	2060	13.2 ± 2.0	12.5 ± 2.7	<0.001
WBC, x10^9/L	2043	6.5 [5.1-8.3]	9.7 [7.3-13]	<0.001
Percent Lymphocytes	2035	16.2 [11.2-22.3]	9.0 [5.9-13]	<0.001
Percent Neutrophils	1973	73 [62-80]	81 [71-86]	<0.001
Neutrophil-Lymphocyte Ratio	1958	4.1 [2.4-13]	8.0 [4.4-13]	<0.001
Platelets, x10^9/L	2049	197 [152-253]	221 [162-292]	<0.001
D-dimer, ug/mL FEU	1268	0.98 [0.61-1.6]	3.1 [1.8-9.6]	<0.001
International Normalized Ratio	1678	1.1 [1.0-1.1]	1.2 [1.1-1.3]	<0.001
APTT, seconds	1665	33 [30-36]	33 [30-38]	0.015
BUN, mg/dL	2066	14 [10-21]	39 [25-65]	<0.001
Creatinine, mg/dL	2066	0.95 [0.75-1.22]	1.64 [1.09-3.10]	<0.001
Bicarbonate, mmol/L	2065	23 [21-25]	21 [17-23]	<0.001
Sodium, mml/L	2066	137 [134-140]	138 [134-143]	<0.001
Troponin-T, ng/L	1825	11 [6-19]	43 [22-98]	<0.001
Interleukin-6, pg/mL	907	22 [6.0-54]	82 [40-157]	<0.001
C-Reactive Protein, mg/L	1900	87 [39-149]	183 [116-276]	<0.001
Lactate Dehydrogenase, U/L	1866	366 [276-481]	516 [391-707]	<0.001
Ferritin, ng/mL	1826	542 [259-1028]	962 [515-181]	<0.001
Erythrocyte Sedimentation Rate, mm/hr	1594	64 [43-85]	88 [65-11]	<0.001
**Vitals**				
Mean Arterial Pressure, mmHg	2076	93 [85-102]	90 [79-100]	<0.001
Temperature, °F	2076	99.6 ± 1.6	98.9 ± 1.5	<0.001

Heart Rate, per min	2075	98 ± 18	98 ± 21	0.826
P_aO_2/F_iO_2 ratio	2055	189 [76-252]	71 [52-162]	<0.001
Respiratory Rate, per min	2073	20 [18-22]	20 [18-25]	<0.001
SOFA	1972	2 [1-6]	4 [3-6]	<0.001
**Outcomes**				
DNR history	2077	47 (3.6)	56 (7.4)	<0.001
Intubation	2077	146 (11)	153 (20)	<0.001
Death	2077	147 (11)	329 (44)	<0.001
Intubation or Death	2077	230 (17)	393 (52)	<0.001
Time to Intubation, hours	299	107 [51.3-207]	72 (43-144)	0.013
Time to Death, hours	476	277 [156-510]	163 (87.1-291)	<0.001
Time to Intubation or Death, days	628	145 (65.5-264]	115 (57.3-202)	0.002

Data are presented as n (%), median [IQR], or mean ± SD.

S17 Table. Characteristics of hospitalized adults with non-critical COVID-19 from the Cornell wild type wave cohort by the 2-class subphenotype model.

	No. available	Hypoinflammatory	Hyperinflammatory	p-value
Number of Patients	1214	695	519	
**Demographics**				
Age, years	1214	66 [53-78]	69 [58-79]	0.003
Male sex	1214	327 (47)	370 (71)	<0.001
Body Mass Index	1107	29 + 7.4	27.6 + 7.0	0.013
Race	1214			0.050
Black		104 (15)	53 (10)	0.019
White		194 (28)	151 (29)	0.699
Other/Unknown		397 (57)	315 (61)	0.234
Hispanic Ethnicity	1214	188 (27)	111 (21)	0.028
**Comorbidities**				
Any Below Comorbidity	1214	533 (77)	422 (81)	0.061
Cancer	1214	204 (29)	139 (27)	0.358
Chronic kidney disease	1214	133 (19)	120 (23)	0.105
Chronic heart failure	1214	107 (15)	99 (19)	0.107
Diabetes (without complications)	1214	294 (42)	193 (37)	0.082
Diabetes (with complications)	1214	174 (25)	154 (30)	0.083
Essential hypertension	1214	434 (62)	333 (64)	0.580
Smoker (current or former)	1214	205 (29)	181 (35)	0.054
Charlson comorbidity Index	1187	2 [1-5]	2 [1-5]	0.731
**Laboratory**				
Albumin, g/dL	1192	3.4 + 0.6	3.0 + 0.5	<0.001
ALT, U/L	1192	26 [17-42]	45 [28-74]	<0.001
Bilirubin, mg/dL	1192	0.5 [0.4-0.7]	0.7 [0.5-1.1]	<0.001
Hemoglobin, g/dL	1214	13 + 2.0	13 + 2.1	0.005
WBC, x10^9/L	1200	5.8 [4.3-7.5]	8.8 [6.6-11]	<0.001
Lymphocyte count, x10^9/L	1185	0.9 [0.6-1.2]	0.70 [0.5-1.0]	<0.001
Percent Neutrophils	782	71 [63-79]	82 [76-88]	<0.001
Platelets, x10^9/L	1211	191 [147-247]	228 [170-303]	<0.001
D-dimer, ug/mL FEU	821	460 [271-835]	686 [383-1771]	<0.001
International Normalized Ratio	1031	1.1 [1.0-1.2]	1.2 [1.1-1.3]	<0.001
APTT, seconds	996	31 [29-34]	32 [29-35]	0.079
BUN, mg/dL	1212	16 [11-23]	22 [15-39]	<0.001
Creatinine, mg/dL	948	0.83 [0.68-1.1]	0.96 [0.77-1.5]	<0.001
Bicarbonate, mmol/L	1212	25 [22-28]	25 [21-27]	0.001
Sodium, mml/L	1212	137 [134-140]	137 [133-140]	0.300
Troponin-T, ng/L	1047	0.03 [0.03-0.03]	0.03 [0.03-0.1]	<0.001
Interleukin-6, pg/mL	115	9.2 [5.0-21]	27 [11-62]	<0.001
C-Reactive Protein, mg/L	915	6.1 [2.9-9.5]	17.4 [11.7-24.3]	<0.001
Lactate Dehydrogenase, U/L	1108	321 [255-399]	518 [421-651]	<0.001
Ferritin, ng/mL	1009	414 [204-801]	1258 [718-2000]	<0.001
Erythrocyte Sedimentation Rate, mm/hr	840	56 [37-80]	82 [60-106]	<0.001
**Vitals**				
Mean Arterial Pressure, mmHg	1214	95 [86-104]	94 [86-103]	0.292
Temperature, °F	1214	37.3 +0.79	37.3 + 0.89	0.630
Heart Rate, per min	1214	94 + 19	100 + 19	<0.001

P_aO_2/F_iO_2 ratio	1214	248 [163-319]	88.6 [60.2-196]	<0.001
Respiratory Rate, per min	1214	18 [18-20]	20 [18-25]	<0.001
**Outcomes**				
Intubation	1214	81 (12)	166 (32)	<0.001
Death	1214	53 (7.6)	93 (18)	<0.001
Intubation or Death	1214	114 (16)	215 (41)	<0.001
Time to Intubation, hours	256	77.6 [50.0-122]	75.1 [43.2-116]	0.408
Time to Death, hours	199	340 [170-215]	214 [112-541]	0.016
Time to Intubation or Death, days	360	117 [59-248]	80.1 [47.3-140]	<0.001

Data are presented as n (%), median [IQR], or mean ± SD.

S18 Table. Characteristics of hospitalized adults with non-critical COVID-19 from the Columbia delta wave cohort by the 2-class subphenotype model

	No. available	Hypoinflammatory	Hyperinflammatory	p-value
Number of Patients	1536	942	594	
**Demographics**				
Age, years	1536	64 [50-76]	70 [59-80]	<0.001
Male sex	1536	459 (49)	326 (55)	0.022
Body Mass Index	1332	29 + 6.9	28 + 6.7	0.008
Race	1536			0.029
Black		152 (16)	91 (15)	0.723
White		260 (28)	131 (22)	0.018
Other/Unknown		530 (56)	372 (63)	0.016
Hispanic Ethnicity	1536	507 (54)	312 (53)	0.657
**Comorbidities**				
Any Below Comorbidity	1536	583 (62)	388 (65)	0.192
Cancer				
Chronic kidney disease	1536	160 (17)	123 (21)	0.078
Chronic heart failure	1536	144 (15)	112 (19)	0.079
Diabetes (without complications	1536	350 (37)	229 (39)	0.620
Diabetes (with complications)	1536	193 (20)	154 (26)	0.016
Essential hypertension	1536	496 (53)	315 (53)	0.927
Smoker (current or former)	1536	142 (15)	66 (11)	0.033
Charlson comorbidity index	1536	1 [0-4]	1 [0-4]	0.302
**Laboratory**				
Albumin, g/dL	1482	3.9 + 0.4	3.5 + 0.5	<0.001
ALT, U/L	1475	25 [17-41]	31 [19-56]	<0.001
Bilirubin, mg/dL	1481	0.4 [0.3-0.6]	0.5 [0.3-0.7]	<0.001
Hemoglobin, g/dL	1533	12.8 + 2.2	12.6 + 2.3	0.046
WBC, $x10^9/L$	1520	5.6 {4.3-7.3]	8.3 [6.3-11.2]	<0.001
Percent Lymphocytes	1467	18 [11-25]	9.0 [4.9-14]	<0.001
Percent Neutrophils	1268	69 [54-76]	78 [60-85]	<0.001
Neutrophil-Lymphocyte Ratio	1258	3.3 [1.7-10.2]	6.0 (2.8-10.2]	<0.001
Platelets, $x10^9/L$	1521	188 [149-242]	232 [169-303]	<0.001
D-dimer, ug/mL FEU	1098	0.88 [0.56-1.5]	1.9 [1.1-3.3]	<0.001
International Normalized Ratio	1180	1.1 [1.0-1.5]	1.1 [1.1-1.2]	<0.001
APTT, seconds	1176	32 [29-35]	34 [30-37]	<0.001
BUN, mg/dL	1531	15 [10-23]	21 [14-35]	<0.001
Creatinine, mg/dL	1533	1.06 [0.83-1.38]	1.19 [0.91-1.74]	<0.001
Bicarbonate, mmol/L	1532	24 [22-26]	23 [20-25]	<0.001
Sodium, mml/L	1533	137 [135-140]	137 [133-140]	0.015
Troponin-T, ng/L	1273	13 [7-24]	21 [11-41]	<0.001
Interleukin-6, pg/mL	397	22.7 [10.7-47.3]	69.8 [35.4-118]	<0.001
C-Reactive Protein, mg/L	1372	49.8 [15.4-89.1]	173 [121-229]	<0.001
Lactate Dehydrogenase, U/L	1148	317 [245-408]	492 [382-655]	<0.001
Ferritin, ng/mL	1207	392 [187-800]	901 [469-1592]	<0.001
Erythrocyte Sedimentation Rate, mm/hr	961	47 [29-65]	83 [61-106]	<0.001
**Vitals**				
Mean Arterial Pressure, mmHg	1533	94 [85-103]	93 [85-102]	0.393
Temperature, ^oF	1533	99.4 + 1.4	99.2 + 1.5	0.007

Heart Rate, per min	1535	95 + 19	100 + 19	<0.001
P_aO_2/F_iO_2 ratio	1518	236 [178-304]	80.5 [50.3-196]	<0.001
Respiratory Rate, per min	1536	18 [18-20]	20 [18-22]	<0.001
SOFA	1302	2 [1-4]	3 [2-4]	<0.001
**Outcomes**				
DNR history	1536	30 (3.2)	33 (5.5)	0.032
Intubation	1536	23 (2.4)	41 (6.9)	<0.001
Death	1536	63 (6.7)	161 (27)	<0.001
Intubation or Death	1536	70 (7.4)	177 (30)	<0.001
Time to Intubation, hours	64	208 [96.9-337]	164 [115-258]	0.532
Time to Death, hours	222	491 [323-908]	355 [199-788]	0.016
Time to Intubation or Death, days	250	346 [218-535]	273 [150-530]	0.055

Data are presented as n (%), median [IQR], or mean ± SD.

S19 Table. Seed sensitivity analysis of the XGBoost subphenotype prediction model.

Seed	Overall Accuracy	Average AUROC	AUROC (1 to rest)	AUROC (2 to rest)	AUROC (3 to rest)
42	0.829	0.958	0.962 (0.954-0.970)	0.952 (0.942-0.961)	0.961 (0.952-0.970)
1616	0.810	0.959	0.950 (0.940-0.960)	0.936 (0.924-0.947)	0.963 (0.954-0.971)
5173	0.822	0.952	0.955 (0.945-0.964)	0.941 (0.930-0.952)	0.962 (0.952-0.971)
7199	0.841	0.962	0.963 (0.955-0.971)	0.954 (0.945-0.964)	0.969 (0.961-0.976)
9180	0.824	0.956	0.960 (0.951-0.968)	0.94 (0.931-0.953)	0.967 (0.959-0.975)

S1 Figure. Study flow diagram for the Columbia wild type wave cohort.

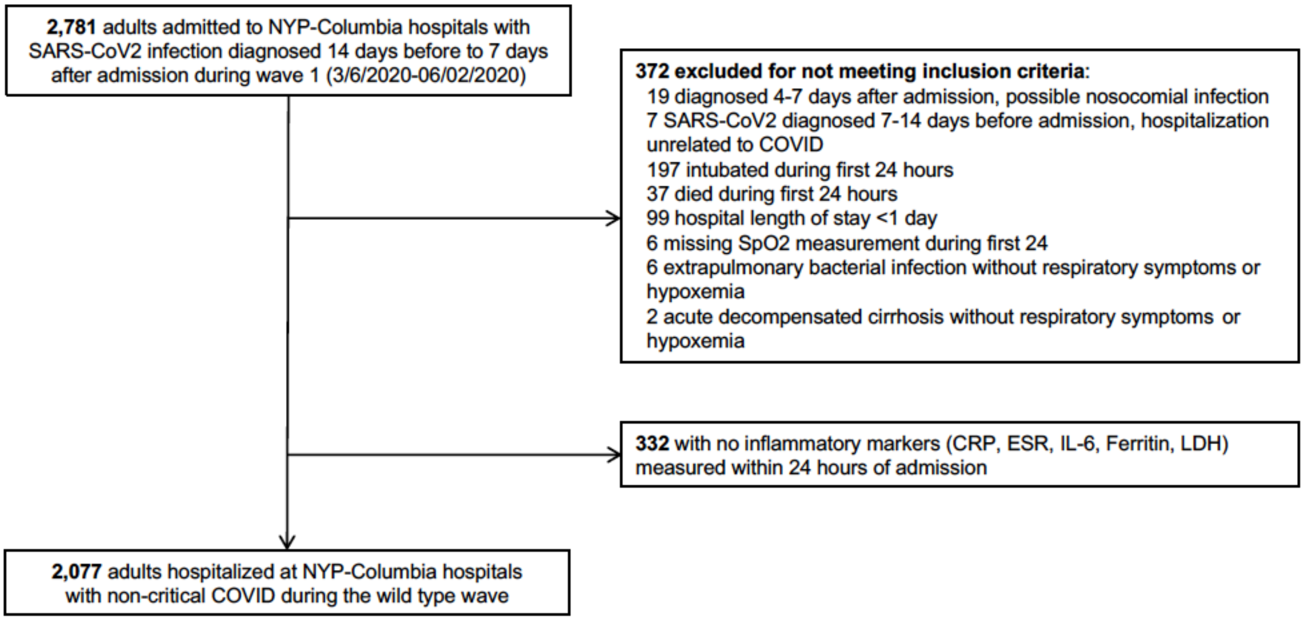

S2 Figure. Study flow diagram for the Cornell wild type wave cohort.

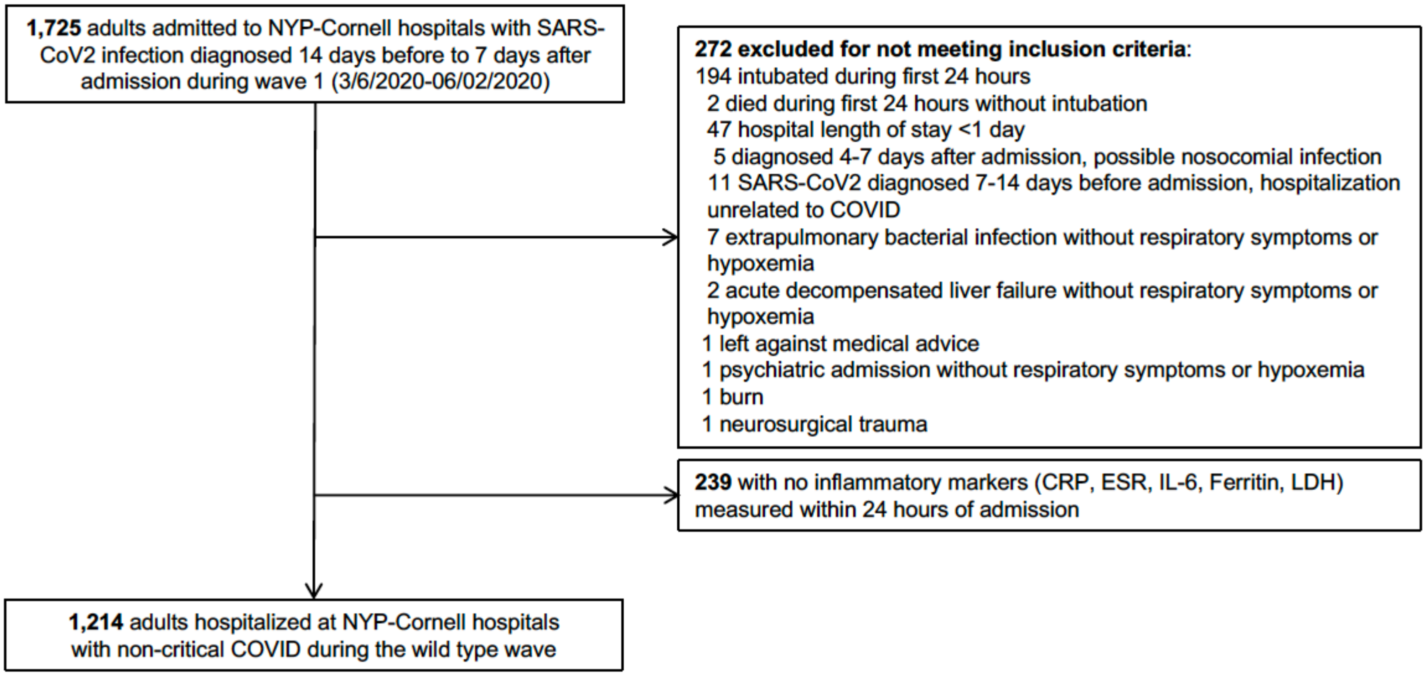

S3 Figure. Study flow diagram for the Columbia wild type wave cohort.

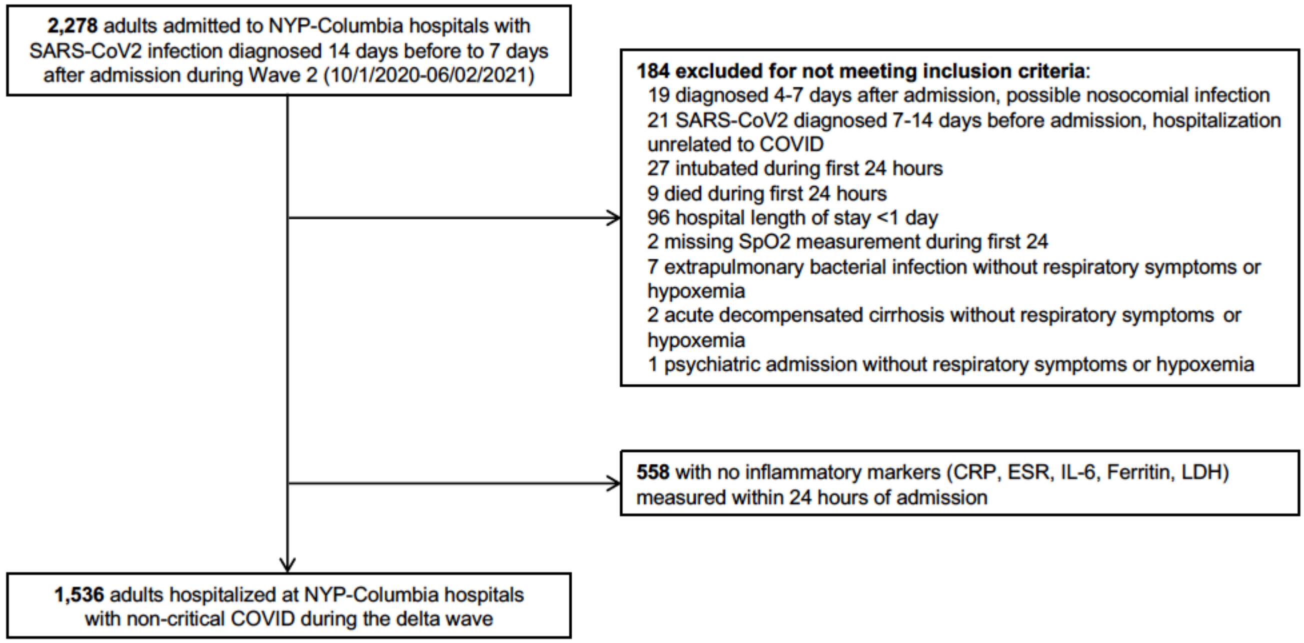

S4 Figure. Dot-box plots of plasma levels of inflammatory biomarkers and D-dimer. Boxes represent the interquartile range, and the middle bar represents the median. P values for each plot are all < 0.001. The upper limit of detection for D-Dimer assay is 20 ug/ml FEU at Columbia and 100 ug/ml at Cornell. To plot Columbia and Cornell data on the same axes, Cornell patient values of >20 ug/ml were assigned a level of 20 mg/ml. Median [IQR] values of each of the biomarkers are listed in Tables E12-E14. IL-6: interleukin-6. CRP: C-reactive protein. LDH: lactate dehydrogenase. ESR: Erythrocyte sedimentation rate.

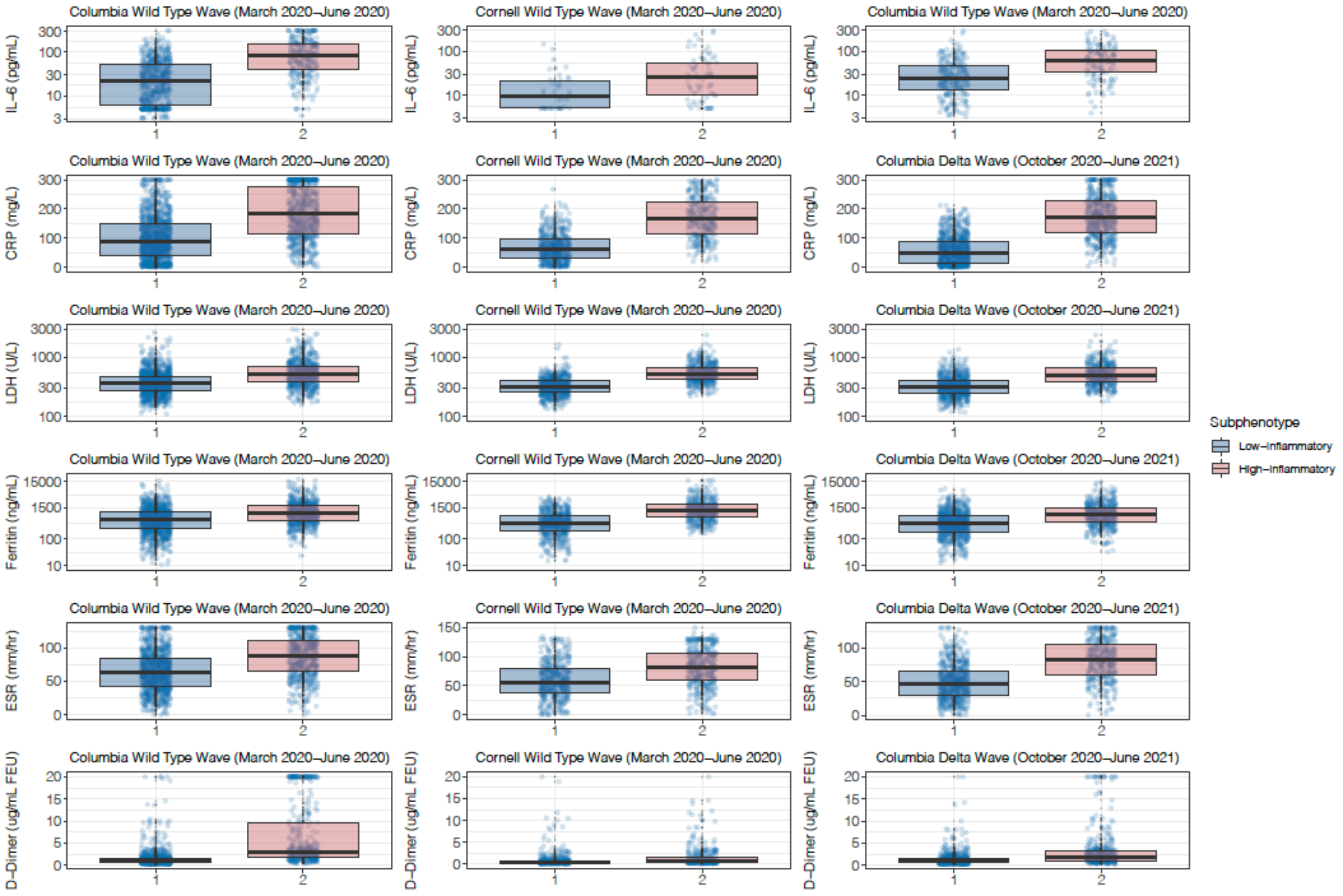

S5 Figure. Kaplan-Meier survival plots right-censored at day 90 stratified by the 2-class latent class model of COVID-19 Acute Lung Injury in the Columbia wild type wave (derivation cohort), the Cornell wild type wave (external validation cohort), and the Columbia delta wave (longitudinal validation cohort). All logrank p <0.001.

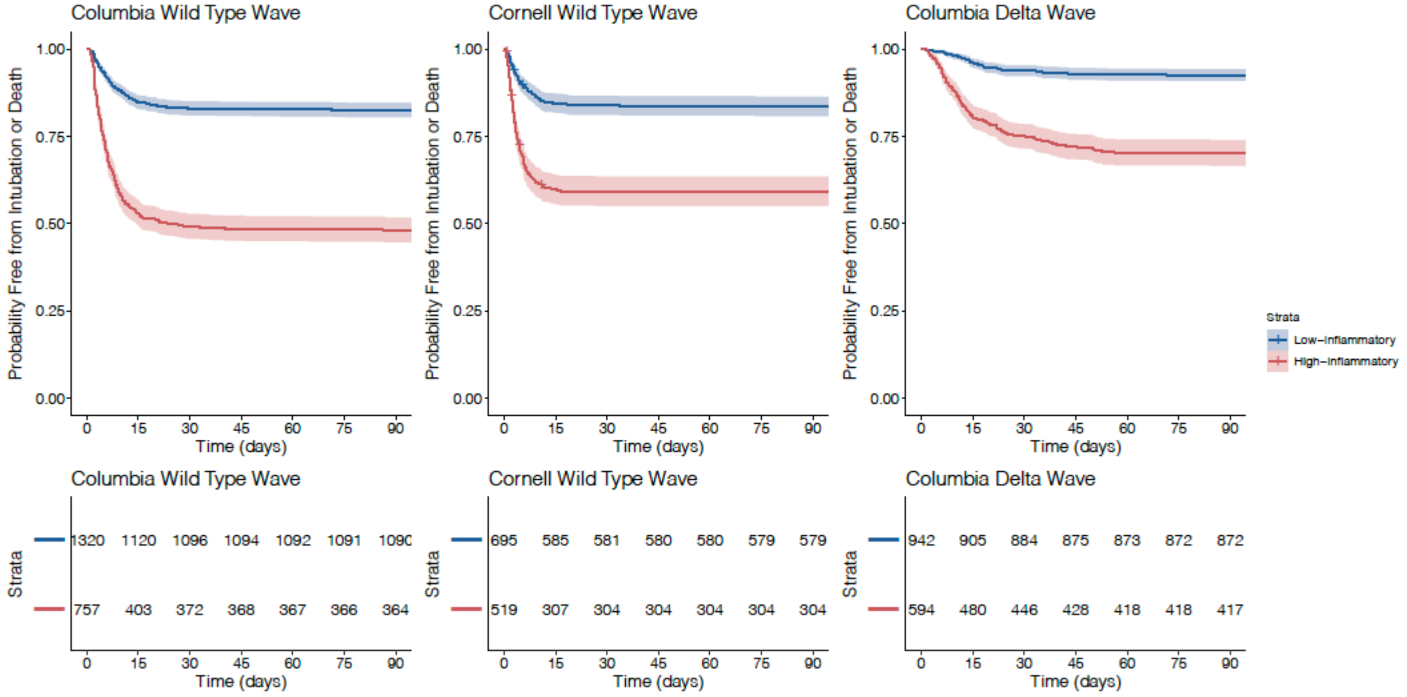

SUPPORTING INFORAMTION REFERENCES

1. Johnson S, Friedman C, Cimino JJ, Clark T, Hripcsak G, Clayton PD. Conceptual data model for a central patient database. *Proc Annu Symp Comput Appl Med Care.* 1991:381-385.

2. Hripcsak G, Duke JD, Shah NH, et al. Observational Health Data Sciences and Informatics (OHDSI): Opportunities for Observational Researchers. *Stud Health Technol Inform.* 2015;216:574-578.

3. Schenck EJ, Hoffman KL, Cusick M, Kabariti J, Sholle ET, Campion TR, Jr. Critical carE Database for Advanced Research (CEDAR): An automated method to support intensive care units with electronic health record data. *J Biomed Inform.* 2021;118:103789.

4. Schenck EJ, Hoffman KL, Oromendia C, et al. A Comparative Analysis of the Respiratory Subscore of the Sequential Organ Failure Assessment Scoring System. *Ann Am Thorac Soc.* 2021;18(11):1849-1860.

5. Cowen ME, Dusseau DJ, Toth BG, Guisinger C, Zodet MW, Shyr Y. Casemix adjustment of managed care claims data using the clinical classification for health policy research method. *Med Care.* 1998;36(7):1108-1113.

6. Sundararajan V, Henderson T, Perry C, Muggivan A, Quan H, Ghali WA. New ICD-10 version of the Charlson comorbidity index predicted in-hospital mortality. *J Clin Epidemiol.* 2004;57(12):1288-1294.

7. Yanez ND, Fu AY, Treggiari MM, Kirsch JR. Oropharyngeal Oxygen Concentration Is Dependent on the Oxygen Mask System and Sampling Location. *Respir Care.* 2020;65(1):29-35.

8. Paul JE, Hangan H, Hajgato J. The OxyMask(™) development and performance in healthy volunteers. *Med Devices (Auckl).* 2009;2:9-17.

9. Geleris J, Sun Y, Platt J, et al. Observational Study of Hydroxychloroquine in Hospitalized Patients with Covid-19. *N Engl J Med.* 2020;382(25):2411-2418.

10. Anderson MR, Geleris J, Anderson DR, et al. Body Mass Index and Risk for Intubation or Death in SARS-CoV-2 Infection : A Retrospective Cohort Study. *Ann Intern Med.* 2020;173(10):782-790.

11. Vital Statistics of New York State Current Data Release Schedule. https://www.health.ny.gov/statistics/vital_statistics/vs_data_release.htm. Accessed June 10, 2023.
